# Exponential super-twisting control for nonlinear systems with unknown polynomial perturbations

**DOI:** 10.1038/s41598-024-53761-2

**Published:** 2024-02-11

**Authors:** Jianghua Liu, Jiang Zhu, Karim Khayati, Dong Zhong, Jinguang Jiang

**Affiliations:** 1 School of Electrical and Information Engineering, Hubei University of Science and Technology, Xianning, Hubei 437100 China; 2 Department of Mechanical and Aerospace Engineering, Royal Military College of Canada, Kingston, ON Canada; 3 National Satellite Positioning System Engineering Technology Research Center, Wuhan University, Wuhan, Hubei China

**Keywords:** Electrical and electronic engineering, Mechanical engineering

## Abstract

The study focuses on the control of nonlinear dynamic systems in the presence of parameter uncertainties, unmodeled dynamics, and external disturbances. The lumped perturbation is assumed to be bounded within a polynomial in the system state with the polynomial parameters and degrees unknown a priori such that it accommodates a quite wider range dynamic systems. Based on the studies in recent super-twisting algorithm designs and the idea from adaptive sliding mode control for nonlinear systems with uncertainties, we propose a novel adaptive super-twisting algorithm with exponential reaching law, or exponential super-twisting algorithm (ESTA), for the high-stability and acceptable accuracy control of the aimed nonlinear dynamics. The stability analysis and practical finite-time (PFT) convergence are proven using Lyapunov theory and an intuitive analysis of the control behaviour. Simulations are performed to compare the proposed ESTA with the existing super-twisting method and the traditional proportional integral differential control. The simulation results demonstrate the effectiveness of the proposed ESTA in terms of the fastest settling time and the smallest overshoot.

## Introduction

During past few decades, sliding mode control (SMC) has gained much attention for its robustness in terms of parameter variations that occur in the control channel and the finite time convergence (FTC) to the sliding surface^1–6^. A high-frequency oscillation called chattering is the well-known drawback of the SMC. To attenuate the chattering phenomenon and improve the accuracy, the high-order SMC (HOSMC) has been studied intensively and shown effectiveness^7,8^. As an important class of HOSMC, the super-twisting algorithm (STA) introduced in^9^ attracts a lot of attention^10–14^. Chalanga et al.^11^ proposed an STA based output feedback stabilization for perturbed double-integrator system. A describing function based STA was presented in^15^. Yan et al., studied the quantization effect^16^ on STA. The reaching time estimation and convergence condition on STA were analysed in^10,12,17^ and^13^, respectively. For systems with saturated control action, Seeber and Reichhartinger investigated conditioned STA^18^. For the systems subject to T-periodic perturbations, Papageorgiou and Edwards^19^ investigate the stability properties and performance of super-twisting sliding-mode control loops. STA techniques were applied to different areas such as Mars entry trajectory tracking with nonsingular terminal sliding mode surface^20^, control of robot manipulators^21,22^ and mobile robots^23^ using robust high-order form, altitude control of a quadrotor unmanned aerial vehicle^24^, adaptive STA control of multi-quadrotor under external disturbance^25^, aircraft at high angle of attack^26^, STA control combined with radial basis function neural network for micro gyroscope^27^, energy management control for integrated DC micro-grid^28^, STA control of passive gait training exoskeleton driven by pneumatic muscles^29^, STA non-sigular fast terminal sliding motor control of interior permanent magnet synchronous motor^30^, and STA state observer based controllers for induction motor drive^14^ and for permanent magnet synchronous motor drive^31,32^ systems. Among these STA techniques, it is assumed that the system uncertainties or external perturbations are bounded within some constants and/or some Lipschitz functions with the boundaries known a priori. To ensure the stability, the system lumped uncertainties should be compensated completely. Consequently, the feedback control gains in STA techniques tend to be overestimated. The overestimated gains may guarantee the system’s stability. However, it may also excite the unmodeled dynamics and/or undesirable chattering^33^.

To overcome the drawback of the overestimation and to deal with uncertainties of unknown bounds, the design of adaptive sliding mode control (ASMC) was introduced^2,34–38^ where the time-varying switching gain is designed to adaptively compensate for the lumped uncertainties. Most ASMC techniques use integral adaptation laws or integral adaptation law combined with other techniques such as the $\sigma$-modification^34^ or the dead zone method^35^. It is shown that, by using integral-type adaptation law, the systems with uncertainties of unknown constant bounds have finite-time stability^36^. However, the system response to the perturbation is relatively slow and, even though the overestimation is avoided somehow, the chattering phenomenon is still observed^39^. To further attenuate the chattering phenomenon, a possible adaptation law is to reduce the switching gain to a minimum admissible value since the magnitude of the chattering level is proportional to the magnitude of the switching gain^37^. In^37^, the ASMC law is applied to STA and uses a low-pass filter to tune the switching gain in the control to a possibly minimum value once the sliding mode is established. However, it still requires a large enough feedback gain to compensate the perturbation with affine function form in the system state, and the use of a low-pass filter introduces a time delay which affects the transient phenomenon.

To achieve fast response and chattering-attenuation property, asymptotic reaching laws rather than ASMC can be found as the form of power reaching law^1^ and an exponential reaching law^40,41^. However, they either lose the robustness when system states are around the sliding surface^1^ or require a priori knowledge of the uncertainty bounds^40,41^. Yang *et al.*^42^ proposed an ASMC technique using exponential reaching law, called adaptive exponential sliding mode control (AESMC), for the control of a beaingless induction motor. Still, for the AESMC design in^42^ all the bounds of the uncertainties must be known a priori. In brief, the aforementioned STA techniques assume that the bounds of the uncertainties are known a priori, while the ASMC techniques assume that the uncertainties are bounded within some constants or affine functions in the system state with the bounds a priori known or unknown. For the case that the bounds of the uncertainties are unknown a priori, seldom researches studied the STA based ASMC. More than the case that the uncertainties are bounded within constants or affine functions in the norm of the system state, the uncertainties may be bounded within some quadratic or cubic functions in the norm of the system state^3^ with the bounds unknown or inaccurate a priori. For instance, with inaccurate parameters the aircraft dynamics and robot manipulators contain uncertainties bounded by quadratic functions of the state. Also, polynomial uncertainties can be found in the systems of the inverted pendulum mounted on a cart, the tunnel-diode circuit dynamics and the Duffing dynamics^3^.

Such cases (with quadratic or cubic uncertainties) represent a wider class of nonlinear systems than the above STA and ASMC techniques considered. More generally, all the above discussed uncertainties can be assumed to be bounded within some polynomials in the norm of the system state with the bounds unknown a priori. To stabilize the above wide class of nonlinear systems, we propose a control method of an exponential super-twisting algorithm (ESTA) where the algorithm structures from STA are integrated with a novel exponential reaching law. Thus, the main contributions of the work can be illustrated as follows.

- Compared to the conventional STA where the bounds of the system’s uncertainties must be known a priori, or the adaptive STA where the uncertainties are assumed to be bounded within some constants or affine functions, a *more general case* of nonlinear systems is considered in this study where the uncertainties are assumed to be bounded within some polynomials in the norm of the system state with the bounds unknown a priori, *i.e.*, both the polynomial parameters and degrees are unknown a priori.
- A novel algorithm, adaptive super-twisting algorithm with exponential reaching law, is proposed to stabilize the aimed nonlinear systems. The stability and the practical finite time convergence^43^ of the new design are proven using Lyapunov theory and an intuitive analysis of the control behavior.
- The proposed new ESTA is compared with the traditional STA and PI methods in simulations, and the simulation results demonstrate the superiority of the new design.

Section “Problem formulation” introduces the nonlinear systems with unknown polynomial uncertainties. The STA technique is recalled with the discussion of the stability issues in some STA designs over past decade. The new ESTA laws are proposed in section “Methodology”. The new design with single-input-single-out (SISO) form is introduced first for the ideal case. Then the multi-input-multi-output (MIMO) forms of the new ESTA are designed for the ideal and real cases. Simulation results are presented in section “Simulation results” to verify the effectiveness of the proposed algorithms. Finally, section “Conclusion” concludes the paper.

## Problem formulation

In this section, we recall the existing STA techniques^9,10,16,26^ to analyze the stability limitations. Useful definition and mathematical lemma are first introduced in this section.

### Definition 1

The signum function is given as

1 $$\begin{aligned} \textrm{sgn}(\sigma ) = \left\{ \begin{array}{rcl} 1 &{} \text {if } \sigma > 0 \\ 0 &{} \text {if } \sigma = 0 \\ -1 &{} \text {if } \sigma < 0 \end{array} \right. \end{aligned}$$

Then,

2 $$\begin{aligned} |\sigma | = \sigma \textrm{sgn}(\sigma ) \end{aligned}$$

### Lemma 1

If the time derivative of $\sigma (t)$ exists, then

3 $$\begin{aligned} \frac{d}{d t} |\sigma (t)| = {\dot{\sigma }} (t) \textrm{sgn}(\sigma (t)) \end{aligned}$$

for all $$\sigma (t) \ne 0$$.

### Problem statement

Consider the uncertain nonlinear dynamic system

4 $$\begin{aligned} \dot{{\sigma }} = f(\sigma , t) + g(\sigma ,t) u \end{aligned}$$

where $\sigma \in \chi \subset {\mathbb {R}}^n$ is the measured signal designating the system state or any sliding variable, $t \in {\mathbb {R}}^+$ is the time, and $u \in {\mathbb {R}}^n$ is the control input signal.

0 is an equilibrium of (4). Function $f(\sigma , t) \subset {\mathbb {R}}^n$ represents lumped perturbation containing parameter uncertainties, unmodeled dynamics, and external disturbances and Function $g(\sigma , t)\subset {\mathbb {R}}^{n \times n}$ contains parameter uncertainties.

#### Assumption 1

The norm of the perturbation ${f}(\sigma , t)$ is upper-bounded with some unknown polynomials in the state vector $\sigma \in \chi$. More specifically,

5 $$\begin{aligned} \Vert {f}(\sigma , t)\Vert \le \sum ^{q}_{i = 0} a_i \Vert \sigma \Vert ^{n_i} \end{aligned}$$

where *q* is an uncertain finite integer, $a_i$ ($i = 0, 1, \cdots , q$) uncertain non-negative finite values, and $n_i$ positive real scalars.

Note, Assumption 1 includes but not limits to the following example.

#### Example 1

6 $$\begin{aligned} \Vert {f}(\sigma , t)\Vert \le a_0 + a_1 \sqrt{\Vert \sigma \Vert } + a_2 \Vert \sigma \Vert + a_3 \Vert \sigma \Vert ^{3/2} + \cdots \end{aligned}$$

#### Assumption 2

Let $g(\sigma , t)^T$ be the transpose matrix of $g(\sigma , t)$. The uncertain term ${g}(\sigma , t)$ is positive definite in wide sense, *i.e.*, its symmetric part $g_s$ defined by

7 $$\begin{aligned} g_s(\sigma , t) = \dfrac{1}{2} \bigg [ g(\sigma , t) + g(\sigma , t)^T \bigg ] > 0 \end{aligned}$$

is positive definite in the regular meaning.

The assumption 2 implies that the minimum eigenvalue of $g_s$ is lower-bounded by a positive finite constant. In other words, there exists a positive finite constant ${\underline{b}}$ such that

8 $$\begin{aligned} g_s(\sigma , t) \ge {\underline{b}}* I_n > 0 \end{aligned}$$

where $I_n$ is an identity matrix with dimension *n*.

#### Remark 1

Uncertainty $f(\sigma , t)$ in *Assumption* 1 takes into account a large class of uncertainties. Many studies^6,36^ assume that the uncertainty $\Vert {f}(\sigma , t)\Vert$ is bounded by a constant *i.e.*, $\Vert {f}(\sigma , t)\Vert \le a_0$ with $a_0$ possibly unknown. Some other researchers^16,34,37^ assume that $\Vert {f}(\sigma , t)\Vert$ is bounded by an affine function in the system state with parameters unknown, *i.e.*, $\Vert {f}(\sigma , t)\Vert \le a_0 + a_1 \Vert \sigma \Vert$ with $a_0$ and $a_1$ possibly unknown. *Assumption* 1 covers all the cases described in^6,16,34,36,37,44^. Moreover, it covers (but not limited to) the case of quadratic uncertainties, *i.e.*, $\Vert {f}(\sigma , t)\Vert \le a_0 + a_1 \Vert \sigma \Vert + a_2 \Vert \sigma \Vert ^2$ with unknown $a_0 > 0$, $a_1 > 0$ and $a_2 > 0$. Thus, the systems under *Assumption*1 represent a large class of nonlinear dynamics systems with uncertainties.

#### Remark 2

Usually the control parameter ${g}(\sigma , t)$ is assumed to be known a priori. For the case where ${g}(\sigma , t)$ is varied, the bounds of the variations usually are assumed known a priori as well. In this contribution, we consider the statement (7), that is, the variation values of the control parameter $g(\sigma , t)$ are loosely structured, which is another way extending the class of nonlinear systems to be addressed.

### Existing super-twisting algorithm based design

For simplicity, we recall the existing STA designs in the scalar case. To steer $\sigma$ to zero, STA techniques^9,10,12,15,16,18,26^ are proposed with the control *u*(*t*) basically defined as

9 $$\begin{aligned} u = - \left( k_1 \sqrt{|\sigma |} \textrm{sgn}(\sigma ) + k_2 \int _0^t \textrm{sgn}(\sigma (\tau )) d \tau \right) / {\underline{b}} \end{aligned}$$

for $k_1$ and $k_2$ sufficiently large. In particularly, $f(\sigma , t)$ in (4) is split into two terms as $f(\sigma , t)= \gamma (\sigma , t) + a(\sigma , t)$ with the following two conditions must be satisfied.^10,12,15,16,18,19^.

10 $$\begin{aligned} |\gamma (\sigma , t)|&\le \delta \sqrt{|\sigma |} \end{aligned}$$

11 $$\begin{aligned} | {\dot{a}}(\sigma , t) |&\le L \end{aligned}$$

The selections of the scalars $k_1$ and $k_2$ in the existing STA designs are based on some stability analyses and have different complicated forms^9,16,26^. Alternatively, $k_1$ and $k_2$ are simply selected^10,11,15,18,19^ as

12 $$\begin{aligned} k_1&> \delta \end{aligned}$$

13 $$\begin{aligned} k_2&> L \end{aligned}$$

In^10,11,15,16,18,19^ it is stated that, if the scalars $k_1$ and $k_2$ are sufficiently large or respectively satisfy (12) and (13), the finite-time stability of the STA design (9) is guaranteed.

The condition (11) contains a possible strict ramp disturbance in the domain of time *t*. Consequently, the magnitude of the ramp disturbance may go to infinity as time elapses. However, most the existing STA control designs are difficult to handle a disturbance with extremely large (or infinity) amplitude. It is possible that some researchers are aware of the inappropriateness or unreality of this hypothesis. The condition (11) is not presented in STA designs of^20,22,45^. Papageorgioua and Edwards^19^ used a restricted assumption, a T-periodic perturbation with (11), to replace the simple (11). Base on the ’T-periodic’ assumption, the authors further demonstrate that under smaller gain conditions ($k_2 < L$), the solutions of the closed loop system converge to a stable limit cycle around the origin as well.

#### Remark 3

Note, the **Assumption** 1 includes the condition (10) but not (11). In real dynamic systems, the condition Eq. (11) which possibly contains a strict ramp disturbance is unnecessary and has little practical significance.

Based on the above discussion, we take **Assumptions**1 and 2 instead of the conditions (10) and (11) in the following new ESTA design.

## Methodology

In this section, we will propose a novel adaptive STA based control law, ESTA design, to expand the traditional STA design by using an exponential reaching law to constrain the system states of (4)–(7) to the vicinity of zero in finite time. The finite time convergence to the vicinity of the equilibrium is also defined as practical finite time stability (PFTS) in^43^ or finite settling time stability (FSTS) in^46^. We start the new ESTA design for the system (4)-(7) for the ideal scalar case. Then we extend the new design to the real case with MIMO form.

### Definition 2

The system (4) is said to be practical finite-time stable (PFTS)^43^ if for all $\sigma _0 \in {\textbf {R}}^n$, there exist $\varepsilon >0$ and $t_F(\varepsilon , \sigma _0) < \infty$, such that $\Vert \sigma (t)\Vert \le \varepsilon$ for all $t \ge t_F$.

### Remark 4

The PFTS means that the state $\sigma$ in (4) converges to the vicinity of the equilibrium in finite time. The finite settling time stability (FSTS) defined in^46^ has a similar meaning.

### ESTA design for SISO systems

We first consider an simple SISO case where the dynamic systems are ideal^9^. Consider the following control law

14 $$\begin{aligned} u = - c_1 \left( e^{\sqrt{|\sigma |}} -1 \right) \textrm{sgn}(\sigma ) - c_2 \int _0^t \textrm{sgn}(\sigma (\tau )) d \tau \end{aligned}$$

for some positive constants $c_1$ and $c_2$.

#### Theorem 1

Consider the scalar system (4)–(7) subject to (14), then the state in (4) has PFTS.

#### Proof

Consider the situation where the system state $\sigma$ is outside of the vicinity of the equilibrium, *i.e.*,

15 $$\begin{aligned} |\sigma | \ge \varepsilon > 0 \end{aligned}$$

for any small positive value of $\varepsilon$. We will prove that the state will converge to the domain $|\sigma | \le \varepsilon$ in finite time. Note from (5)

16 $$\begin{aligned} |f&(\sigma , t)| - {\underline{b}} c_1 \left( e^{\sqrt{|\sigma |}} - 1 \right) \nonumber \\&= |f(\sigma , t)| + {\underline{b}} c_1 - {\underline{b}} c_1 e^{\sqrt{|\sigma |}} \nonumber \\&\le {\underline{b}} c_1 + \sum ^{q}_{i = 0} a_i | \sigma |^{n_i} - {\underline{b}} c_1 e^{\sqrt{|\sigma |}} \end{aligned}$$

Since the exponential term ${\underline{b}} c_1 e^{\sqrt{|\sigma |}}$ will eventually be bigger than the polynomial term ${\underline{b}} c_1 + \sum ^{q}_{i = 0} a_i | \sigma |^{n_i}$ as $\sigma$ increasing, the term $|f(\sigma , t)| - {\underline{b}} c_1 \left( e^{\sqrt{|\sigma |}} - 1 \right)$ in (16) is upper bounded, *i.e.*,

17 $$\begin{aligned} |f(\sigma , t)| - {\underline{b}} c_1 \left( e^{\sqrt{|\sigma |}} - 1 \right) \le c^* \end{aligned}$$

for a positive constant $c^*$. Now we consider the time derivative of $|\sigma |$. Using Lemma 1, we obtain for $|\sigma | \ge \varepsilon$,

18 $$\begin{aligned} \frac{d}{d t} |\sigma |&= f(\sigma , t) \textrm{sgn}(\sigma ) + g(\sigma , t) \textrm{sgn}(\sigma ) u \nonumber \\&= f(\sigma , t) \textrm{sgn}(\sigma ) - g(\sigma , t) \textrm{sgn}(\sigma ) c_1 \left( e^{ \sqrt{|\sigma |}} - 1 \right) \textrm{sgn}(\sigma ) \nonumber \\&\hspace{4mm} - g(\sigma , t)\textrm{sgn}(\sigma ) c_2 \int _0^t \textrm{sgn}(\sigma (\tau )) d \tau \nonumber \\&= f(\sigma , t) \textrm{sgn}(\sigma ) - g(\sigma , t) c_1 \left( e^{\sqrt{|\sigma |}} - 1 \right) \nonumber \\&\hspace{4mm} - c_2g(\sigma , t)\textrm{sgn}(\sigma ) \int _0^t \textrm{sgn}(\sigma (\tau )) d \tau \end{aligned}$$

Now we consider two cases of $\sigma$. Case I, the system state is in the positive space of the vicinity of the equilibrium, *i.e.*, $\sigma \ge \varepsilon > 0$. We obtain from (17) and (18),

19 $$\begin{aligned} \frac{d}{d t} |\sigma |&\le |f(\sigma , t)| - {\underline{b}} c_1 \left( e^{\sqrt{|\sigma |}} - 1 \right) - c_2 {\underline{b}} \cdot 1 \int _0^t 1 d \tau \nonumber \\&\le c^* - c_2 {\underline{b}} \int _0^t 1 d \tau \nonumber \\&\le c^* - c_2 {\underline{b}} \int _0^{t^*} 1 d \tau - c_2 {\underline{b}} \int _{t^*}^t 1 d \tau \end{aligned}$$

Let $t^* = c^*/{c_2 {\underline{b}}}$. Then, after time $t^*$ ($t \ge t^*$),

20 $$\begin{aligned} \frac{d}{d t} |\sigma |&\le - c_2 {\underline{b}} \int _{t^*}^t 1 d \tau \nonumber \\&\le - c_2 {\underline{b}}(t - t^*) \end{aligned}$$

Noting that $c_2 {\underline{b}}(t - t^*)$ is continuously increasing as time elapses after $t \ge t^*$. That is, the rate of the decline of the state $|\sigma |$ is getting faster and faster as time elapses and the state $|\sigma |$ will eventually reach the region $|\sigma | \le \varepsilon$ in finite time. By integrating (20) between $t^*$ and $t > t^*$ and using the Comparison Lemma^3^, we obtain

$$\begin{aligned} |\sigma (t)| - |\sigma (t^*)|&\le - \dfrac{1}{2} c_2 {\underline{b}} (t - t^*)^2 \end{aligned}$$

Then the reaching time $t_F$ is estimated as

21 $$\begin{aligned} \varepsilon - |\sigma (t^*)|&\le - \dfrac{1}{2} c_2 {\underline{b}} (t_F - t^*)^2 \nonumber \\ t_F&\le \sqrt{2 \dfrac{|\sigma (t^*)| - \varepsilon }{ c_2 {\underline{b}}} } + t^* \end{aligned}$$

Case II, the system state is in the negative space of the vicinity of the equilibrium, *i.e.*, $\sigma \le - \varepsilon < 0$. We obtain from (17) and (18),

22 $$\begin{aligned} \frac{d}{d t} |\sigma |&\le |f(\sigma , t)| - {\underline{b}} c_1 \left( e^{\sqrt{|\sigma |}} - 1 \right) - c_2 {\underline{b}} \cdot (-1) \int _0^t (-1) d \tau \nonumber \\&\le c^* - c_2 {\underline{b}} \int _0^{t^*} 1 d \tau - c_2 {\underline{b}} \int _{t^*}^t 1 d \tau \end{aligned}$$

Note that the inequality (22) has a same form as (20) in Case I. We conclude that $|\sigma |$ will eventually reach the region $|\sigma | \le \varepsilon$ in finite time and remains on it thereafter with the reaching time estimated as $t_F \le \sqrt{2 \dfrac{|\sigma (t^*)| - \varepsilon }{ c_2 {\underline{b}}} } + t^*$ $\square$

Theorem 1 shows that for any positive constants $c_1$ and $c_2$ the control (14) will always steer the system state to the vicinity of the equilibrium point in finite time even if the lumped perturbation contains some polynomial form in the system state and even if we do not known the boundaries of the uncertainties a priori. The reaching time or the finite settling time $t_F$ of Theorem 1 contains two parts, the compensating time $t^*$ and the converging time $\sqrt{2 \dfrac{|\sigma (t^*)| - \varepsilon }{ c_2 {\underline{b}}}}$. One can see that the settling time $t_F$ depends on the control gain $c_2$ and system parameter ${\underline{b}}$. Generally, $t_F$ can be reduced by increasing $c_2$ and ${\underline{b}}$ values. An insight discussion of the finite settling time $t_F$ estimation can be found in Ref.^39^.

One drawback of the conventional sliding mode control is the chattering phenomenon where the switching function $\textrm{sgn}(\cdot )$ is the main source of it. In real implementation, the function is usually replaced by some smooth functions to attenuate the chattering effects^2,3,37^. In this work, a simple smooth function $\textrm{sgn}(\sigma ) \approx \dfrac{\sigma }{|\sigma | + \mu }$ with a small positive scalar $\mu$^47^ can be used to replace the switching function.

23 $$\begin{aligned} u = - c_1 \left( e^{\sqrt{|\sigma |}} - 1 \right) \dfrac{\sigma }{|\sigma | + \mu } - c_2 \int _0^t \dfrac{\sigma }{|\sigma | + \mu } d \tau \end{aligned}$$

where the small positive constant $\mu$ is related to the thickness of the boundary layer of the real sliding mode^3^. Using the aforementioned small positive scalar $\varepsilon$ as the vicinity of the equilibrium, we have the following proposition.

#### Proposition 1

Consider the scalar system (4)–(7). If the control law is selected as (23), then the states in (4) has PFTS.

The proof is similar to the proof of Theorem 1 and is shown in Appendix “Proof of the Proposition 1”.

#### Remark 5

The smoothing function can be used in most sliding mode control. The main imperfection of the smoothing function is that it is not suitable for direct application of a small number of SMC controls, such as the STA design (9). In other words, when the smoothing function $\textrm{sgn}(\sigma ) \approx \dfrac{\sigma }{|\sigma | + \mu }$ is chosen to approximate the switching function, the existing STA design (9) with conditions (10) and (11) may encounter stability issues which can be seen in the following example.

#### Example 2

**Figure 1:**
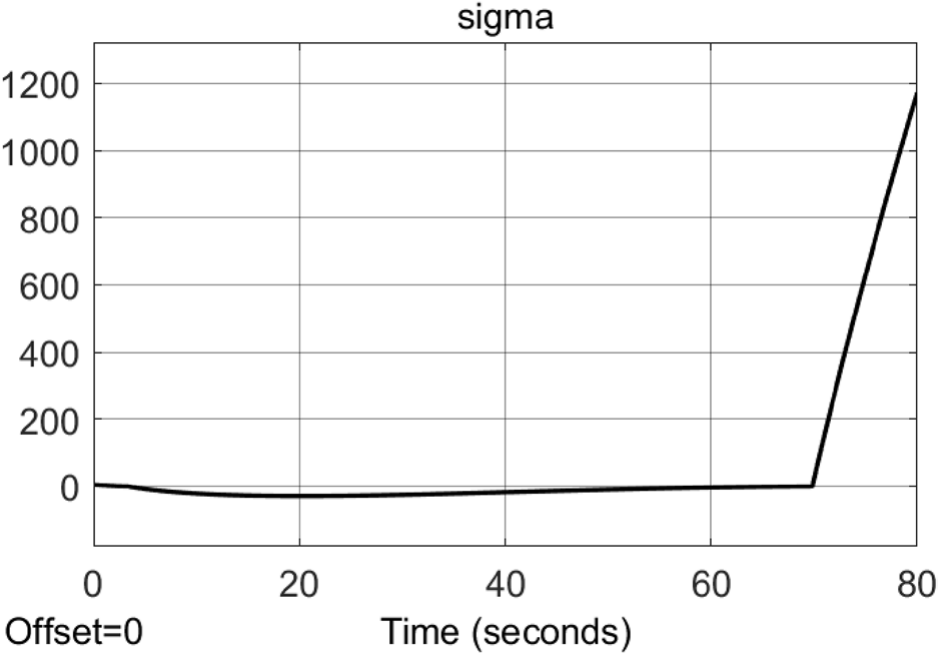
Simulation results of the Example (2) by using the existing STA control (9) with the switching function $\textrm{sgn}(\sigma )$ replaced by $\dfrac{\sigma }{|\sigma | + 0.001}$ for the case I $k_2 = 1.1$.

**Figure 2:**
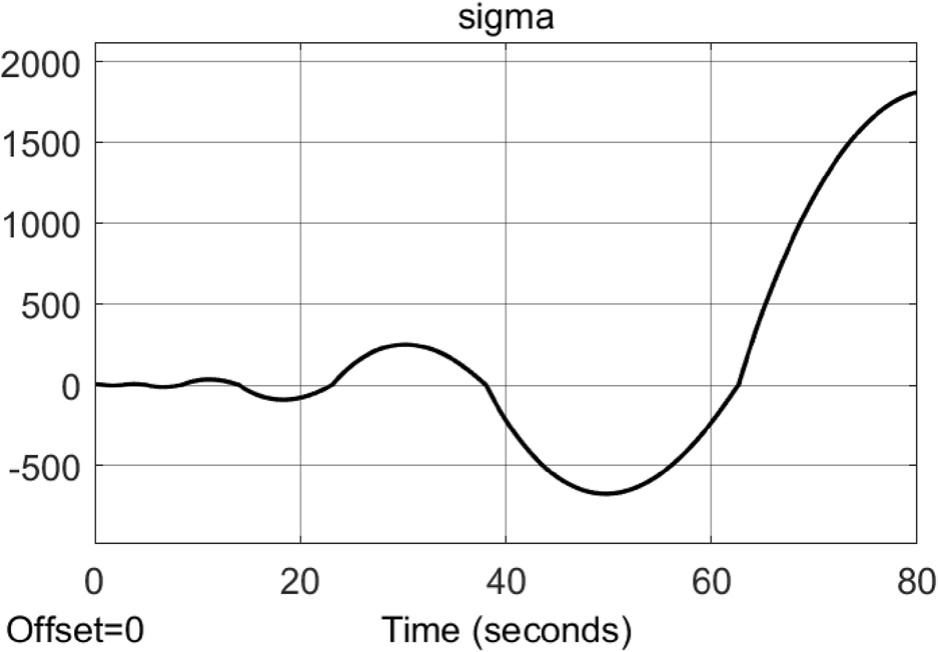
Simulation results of the Example (2) by using the existing STA control (9) with the switching function $\textrm{sgn}(\sigma )$ replaced by $\dfrac{\sigma }{|\sigma | + 0.001}$ for the case II $k_2 = 10$.

Consider the uncertain nonlinear system 4 with the conditions (10) and (11). Let $g(\sigma , t) = {\underline{b}} = 1$, $\gamma (\sigma , t)=0$ and ${\dot{a}}(\sigma , t) = L = 1$ for simplicity. Consider the existing STA control (9) with the switching function $\textrm{sgn}(\sigma )$ replaced by $\dfrac{\sigma }{|\sigma | + \mu }$ where $\mu = 0.001$. Let the designed parameter $k_1 = 1$ satisfying (12). We choose case I $k_2 = 1.1$ to satisfy (13) and case II $k_2 = 10$ to be sufficiently large. The simulation results with sampling time 0.001*s* are shown in Figs. 1 and 2.

The above example shows that by using the existing STA design, if the switching function is approximated by some smoothing functions, the system may become unstable no matter the gain $k_2$ satisfies (13) or $k_2$ is sufficient large. (See Fig. 1 for $k_2 = 1.1$ satisfying (13) and Fig. 2 for $k_2 = 10$ sufficiently large.)

### ESTA for MIMO systems

Now we extend the new ESTA design for the MIMO systems (4)–(7). Let $\Vert \sigma \Vert$ be the 2-norm of the system state $\sigma$. Consider the following control law

24 $$\begin{aligned} u = - c_1 \left( e^{\sqrt{\Vert \sigma \Vert }} -1 \right) \dfrac{\sigma }{\Vert \sigma \Vert } - c_2 \big ( \int _0^t \Vert \sigma \Vert d \tau \big ) \dfrac{\sigma }{\Vert \sigma \Vert } \end{aligned}$$

for some positive constants $c_1$ and $c_2$.

#### Theorem 2

Consider the MIMO system (4)–(7) subject to (24), then the state in (4) has PFTS.

#### Proof

In the following, the argument *t* of most given vector field (*i.e.*, $\sigma$, *f*, *g*, *etc.*) will be omitted for simplicity. Consider the following Lyapunov function candidate

25 $$\begin{aligned} V = \sigma ^T \sigma \end{aligned}$$

Let $u^T$ be the transpose vector of *u* in (24). Using (4) and (24), the time derivative of *V* along the system trajectories is

26 $$\begin{aligned} {\dot{V}}&= {\dot{\sigma }}^T \sigma + \sigma ^T {\dot{\sigma }} \nonumber \\&= \left( f^T + u^T g^T \right) \sigma + \sigma ^T (f + g u) \nonumber \\&= f^T \sigma + \sigma ^T f + u^T g^T \sigma + \sigma ^T g u \end{aligned}$$

Note, the scaler $$f^T \sigma = \left( f^T \sigma \right) ^T =\sigma ^T f$$, then $$f^T \sigma + \sigma ^T f = 2 \sigma ^T f$$. Since $$ c_1$$, $$ c_2$$, $$\Vert \sigma \Vert $$, $$ \big ( e^{\sqrt{\Vert \sigma \Vert }} -1 \big )$$, and $$ \big ( \int _0^t \Vert \sigma \Vert d \tau \big )$$ are scalars, and $$u^T = - c_1 \big ( e^{\sqrt{\Vert \sigma \Vert }} -1 \big ) \dfrac{\sigma ^T}{\Vert \sigma \Vert } - c_2 \big ( \int _0^t \Vert \sigma \Vert d \tau \big ) \dfrac{\sigma ^T}{\Vert \sigma \Vert }$$, we have

27 $$\begin{aligned} u^T g^T \sigma + \sigma ^T g u&= \Big (- c_1 \bigg (e^{\sqrt{\Vert \sigma \Vert }} - 1 \bigg ) \dfrac{\sigma ^T}{\Vert \sigma \Vert } - c_2 \bigg ( \int ^t_0 \Vert \sigma (\tau )\Vert d \tau \bigg ) \dfrac{\sigma ^T}{\Vert \sigma \Vert } \Bigg ) g^T \sigma \nonumber \\&\hspace{3mm} + \sigma ^T g \Bigg (- c_1 \big (e^{\sqrt{\Vert \sigma \Vert }} - 1 \bigg ) \dfrac{\sigma }{\Vert \sigma \Vert } - c_2 \bigg ( \int ^t_0 \Vert \sigma (\tau )\Vert d \tau \bigg ) \dfrac{\sigma }{\Vert \sigma \Vert } \Bigg ) \nonumber \\&= - c_1 \bigg (e^{\sqrt{\Vert \sigma \Vert }} - 1 \bigg ) \dfrac{\sigma ^T}{\Vert \sigma \Vert }g^T \sigma - c_2 \bigg ( \int ^t_0 \Vert \sigma (\tau )\Vert d \tau \bigg ) \dfrac{\sigma ^T}{\Vert \sigma \Vert } g^T \sigma \nonumber \\&\hspace{3mm} - c_1 \bigg (e^{\sqrt{\Vert \sigma \Vert }} - 1 \bigg ) \sigma ^T g \dfrac{\sigma }{\Vert \sigma \Vert } - c_2 \bigg ( \int ^t_0 \Vert \sigma (\tau )\Vert d \tau \bigg ) \sigma ^T g \dfrac{\sigma }{\Vert \sigma \Vert } \nonumber \\&= - c_1 \bigg (e^{\sqrt{\Vert \sigma \Vert }} - 1 \bigg ) \dfrac{\sigma ^T(g^T + g)\sigma }{\Vert \sigma \Vert } - c_2 \bigg ( \int ^t_0 \Vert \sigma (\tau )\Vert d \tau \bigg ) \dfrac{\sigma ^T(g^T + g)\sigma }{\Vert \sigma \Vert } \end{aligned}$$

Then, we obtain from (7), (26) and (27),

28 $$\begin{aligned} {\dot{V}}&= 2 \sigma ^T f - 2 c_1 \bigg (e^{\sqrt{\Vert \sigma \Vert }} - 1 \bigg ) \dfrac{\sigma ^T g_s \sigma }{\Vert \sigma \Vert } - 2 c_2 \bigg ( \int ^t_0 \Vert \sigma (\tau )\Vert d \tau \bigg ) \dfrac{\sigma ^T g_s \sigma }{\Vert \sigma \Vert } \end{aligned}$$

Consider the situation where the system state $\sigma$ is outside of the vicinity of the equilibrium, *i.e.*, $\Vert \sigma \Vert \ge \varepsilon$. Using $\Vert \sigma \Vert = \sqrt{V}$, $\Vert \sigma \Vert ^2 = \sigma ^T \sigma$, and recalling Assumption 2, we reformulate (28) as,

29 $$\begin{aligned} \dfrac{{\dot{V}}}{2\sqrt{V}}&= \dfrac{\sigma ^T}{\Vert \sigma \Vert } f - c_1 \bigg (e^{\sqrt{\Vert \sigma \Vert }} - 1 \bigg ) \dfrac{\sigma ^T g_s \sigma }{\sigma ^T \sigma } \nonumber \\&\hspace{3mm} - c_2 \bigg ( \int ^t_0 \Vert \sigma (\tau )\Vert d \tau \bigg ) \dfrac{\sigma ^T g_s \sigma }{\sigma ^T \sigma } \nonumber \\&\le \Vert f\Vert - c_1 {\underline{b}}\bigg (e^{\sqrt{\Vert \sigma \Vert }} - 1 \bigg ) - c_2 {\underline{b}} \bigg ( \int ^t_0 \Vert \sigma (\tau )\Vert d \tau \bigg ) \end{aligned}$$

We denote by $h(\Vert \sigma \Vert ) = \Vert f\Vert - c_1 {\underline{b}}\bigg (e^{\sqrt{\Vert \sigma \Vert }} - 1 \bigg )$. One can see that $h(\Vert \sigma \Vert )$ is upper-bounded since a positive exponential function ultimately grow faster than any polynomial. That is, there exists a finite scalar ${\overline{h}}$ such that

30 $$\begin{aligned} h(\Vert \sigma \Vert )&\le \sum ^{q}_{i = 0} a_i \Vert \sigma \Vert ^{n_i} + c_1 {\underline{b}} - c_1 {\underline{b}} e^{\sqrt{\Vert \sigma \Vert }} \le {\overline{h}} \end{aligned}$$

for all $\sigma$. Note that $\dfrac{d}{d t} \Vert \sigma \Vert \equiv \dfrac{{\dot{V}}}{2\sqrt{V}}$ for $\Vert \sigma \Vert \ge \varepsilon$. The inequality (29) can be rewritten as

31 $$\begin{aligned} \dfrac{d}{d t} \Vert \sigma \Vert \le {\overline{h}} - c_2 {\underline{b}} \int ^t_0 \Vert \sigma (\tau )\Vert d \tau \end{aligned}$$

For any $\Vert \sigma \Vert \ge \varepsilon$, the term $c_2 {\underline{b}} \int ^t_0 \Vert \sigma (\tau )\Vert d \tau$ in (31) keeps increasing and will eventually compensates for ${\overline{h}}$. Since this compensating action occurs for any $\Vert \sigma \Vert \ge \varepsilon$, we conclude that there exists a time instant $t^*$ such that ${\overline{h}} \le c_2 {\underline{b}} \int ^{t^*}_0 \Vert \sigma (\tau )\Vert d \tau$. Then, after the time instant $t^*$, *i.e.*, $t \ge t^*$, $\int ^t_0 \Vert \sigma (\tau )\Vert d \tau = \int ^{t^*}_0 \Vert \sigma (\tau )\Vert d \tau +\int ^t_{t^*} \Vert \sigma (\tau )\Vert d \tau$. We obtain from (31)

32 $$\begin{aligned} \dfrac{d}{d t} \Vert \sigma \Vert \le - c_2 {\underline{b}} \int ^t_{t^*} \Vert \sigma (\tau )\Vert d \tau \end{aligned}$$

By applying the mojorant curve approach^39^, we conclude that $$\Vert \sigma \Vert 
$$ reaches the region $$\Vert \sigma \Vert \le \varepsilon $$ in finite time and remains on it thereafter with the reaching time estimated as $$t_F \le \dfrac{\pi }{2\sqrt{c_2 {\underline{b}}}} + t^*$$ $$\square $$

The above ESTA algorithm (24) is designed for the ideal case. For the real implementation, the magnitude of the integral term $\int _0^t \Vert \sigma (\tau )\Vert d \tau$ may rise to a very large value since it keeps growing for any $\Vert \sigma \Vert \ne 0$. Also, the term $\Vert \sigma \Vert$ as a denominator in (24) may cause some singularity problem when $\Vert \sigma \Vert$ is close to zero. To eliminate these problems, we use $\varepsilon$-adaptation^36^ and a smoothing function^47^ in the following ESTA design for the MIMO systems (4)–(7) in real implementation.

33 $$\begin{aligned} u&= - c_1 \left( e^{\sqrt{\Vert \sigma \Vert }} -1 \right) \dfrac{\sigma }{\Vert \sigma \Vert + \mu } \nonumber \\&- c_2 \int _0^t (\Vert \sigma (\tau )\Vert - \varepsilon ) d \tau \dfrac{\sigma }{\Vert \sigma \Vert + \mu } \end{aligned}$$

where $c_1$ and $c_2$ are positive gains and $\mu$ is the smoothing factor in the smoothing function $\dfrac{\sigma }{\Vert \sigma \Vert + \mu }$ used for replacing the switching function $\textrm{sgn}(\cdot )$.

#### Proposition 2

Consider the MIMO system (4)–(7) subject to (33), then the states in (4) have PFTS.

The proof is ignored here to save the space.

#### Remark 6

Note that the controllers ESTA (14) and STA (9) use full state feedback control. These controllers can be applied to higher-order systems if they can be converted to first-order systems and the state of the first-order system is available. If the state of the system cannot be measured directly, but the system is observable, it is often possible to construct an “observer” or simply use some differentiator to estimate the full state. When the “observer” or differentiator are good to use, the two-part structure of proposed ESTA methods (14), (23), (24) and (33) can be extended to three-part structure. For example, ESTA controller (14) can be extended to a controller containing a differential term.

34 $$\begin{aligned} u = - c_1 \left( e^{\sqrt{|\sigma |}} -1 \right) \textrm{sgn}(\sigma ) - c_2 \int _0^t \textrm{sgn}(\sigma (\tau )) d \tau - c_3 \left( e^{\dot{\hat{{|\sigma |}}}} -1 \right) \textrm{sgn}(\dot{\hat{{|\sigma |}}}) \end{aligned}$$

where $\dot{\hat{{|\sigma |}}}$ represents the estimation of the differential of the measured sensor signal $\sigma$ if ${\dot{\sigma }}$ is not directly available. The stability proof of the control (34) is verbose and ignored here.

The selection of gains can refer to the gain adjustments of PID control. Generally, the exponential gain $c_1$ in proposed ESTA methods can be first tuned to a relatively large value. Then gain $c_2$ can be tuned to eliminate the steady error. If the measured signals are differentially reliable, $c_3$ can also be tuned to further reduce the overshoot.

#### Remark 7

The reference signal of the controllers designed in this paper can be time-invariant, such as coordinate values, or time-varying, such as trajectories. For the trajectory reference signal, we require it to be second-order differentiable such that the error dynamics equation of the trajectory tracking control system can be constructed.

## Simulation results

Although the STA algorithm appeared in the 90s of the last century, it is still widely used today, such as the literatures^13,14,19,25,29–32^ of the last three years. The core structure of the STA algorithms used in these literatures is consistent. As a result, these newer STA control methods also share common features, such as a reduced settling time, but a decrease in robustness. Compared to the STA methods, the advantage of the ESTA proposed in this paper is that it maintains a short or shorter settling time, while at the same time has a significant improvement in robustness. Because of the comparison in terms of settling time (stability time) and robustness, we chose two quantitative indicators, settling time $t_s$ and overshoot *O*.*S*., for stability analysis.

### Illustrated example

In this section, an example which contains external perturbations and polynomial uncertainties in the norm of the state is given to illustrate the system response of the proposed ESTA. Consider the system (4) with $g(t) = {\underline{b}} = 1$ for simplicity. The lumped perturbation $f(\sigma , t) = f_1(t) + f_2(\sigma )$ is chosen as

35a $$\begin{aligned} f_1(t)&= \alpha _1 \end{aligned}$$

35b $$\begin{aligned} f_2(\sigma )&= \alpha _2 \cdot \sigma + \alpha _3 \left( \sigma ^2 + \sigma ^3 \right) \end{aligned}$$

where $f_1(t)$ represents external disturbances and $f_2(\sigma )$ contains system parameter uncertainties or unmodelled dynamics. In particular, $\alpha _3 (\sigma ^2 + \sigma ^3)$ represents higher order polynomial bounded disturbances if the unknowm scalar $\alpha 3 \ne 0$. In simulation the proposed ESTA control (23) (for real case) is compared to the existing STA control (9) and the conventional proportional-integral (PI) control which has the following form:

36 $$\begin{aligned} u_{PI} = - d_1 \sigma - d_2 \int _0^t \sigma (\tau ) d \tau \end{aligned}$$

Note, the three methods have similar proportional-integral forms. For simplicity and comparability, the ‘P-parameters’ in (9), (23) and (36) are selected as $k_1 = c_1 = d_1 = 1$, as well as the ‘I-parameters’ $k_2 = c_2 = d_2 = 1$. We choose the smoothing factor $\mu = 0.001$ and keep it unchanged for all simulations.

#### Transient response under external disturbances

To test the system transient response under various external disturbances, we let the parameter of the unmodelled dynamics $\alpha _2 = \alpha _3 = 0$ be fixed. Two levels of the external disturbance, $\alpha _1 = 2$ and $\alpha _1 = 5$, are used to respectively represent a moderate and a relatively large external disturbances.

**Figure 3:**
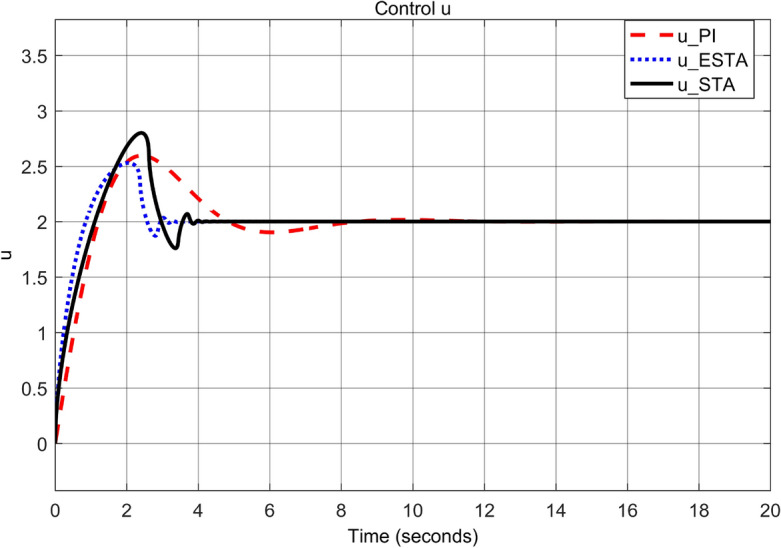
Transient response of the nonlinear system (4) under an external disturbance (35) with $\alpha _1 = {\textbf {2}}$, $\alpha _2 = \alpha _3 = 0$: Control inputs *u*(*t*) using PI (36) (dash red), ESTA (23) (dot blue), and STA (9) (solid black).

**Figure 4:**
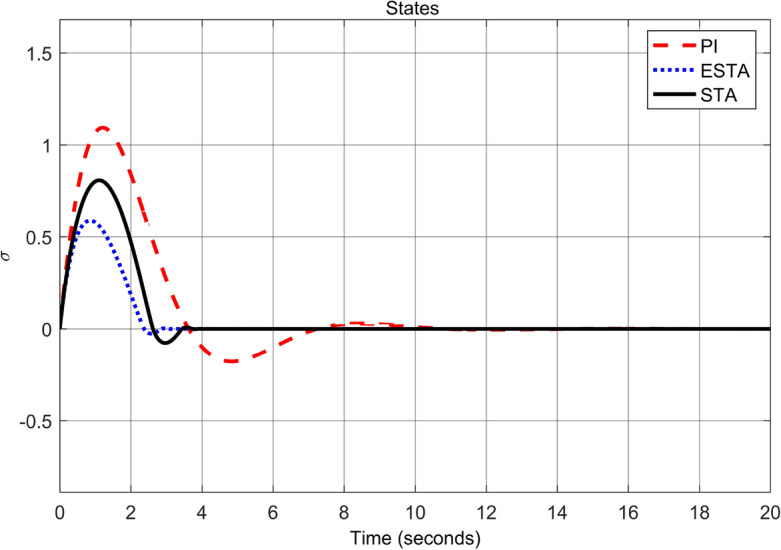
Transient response of the nonlinear system (4) under an external disturbance (35) with $\alpha _1 = {\textbf {2}}$, $\alpha _2 = \alpha _3 = 0$: State $\sigma (t)$ using PI (36) (dash red), ESTA (23) (dot blue), and STA (9) (solid black).

**Figure 5:**
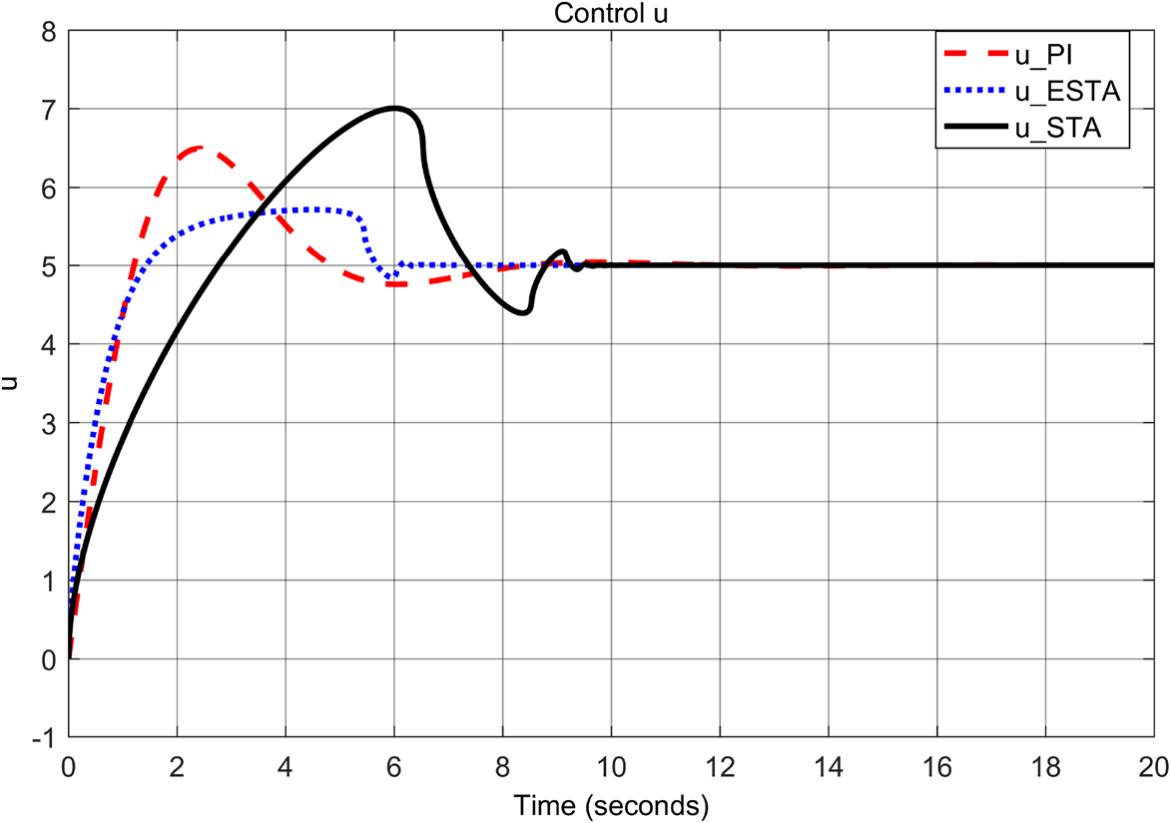
Transient response of the nonlinear system (4) under an external disturbance (35) with $\alpha _1 = {\textbf {5}}$, $\alpha _2 = \alpha _3 = 0$: Control inputs *u*(*t*) using PI (36) (dash red), ESTA (23) (dot blue), and STA (9) (solid black).

**Figure 6:**
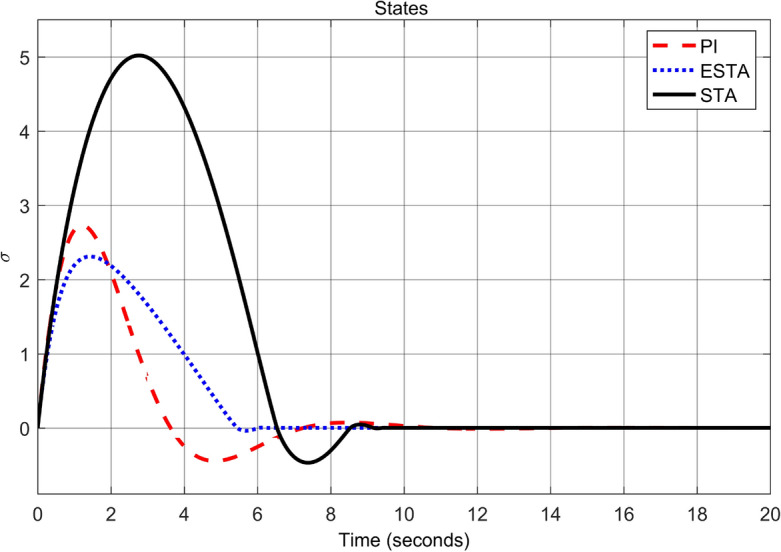
Transient response of the nonlinear system (4) under an external disturbance (35) with $\alpha _1 = {\textbf {5}}$, $\alpha _2 = \alpha _3 = 0$: State $\sigma (t)$ using PI (36) (dash red), ESTA (23) (dot blue), and STA (9) (solid black).

We first choose a moderate external disturbance with $\alpha _1 = 2$ where the simulation results of the state $\sigma (t)$ and the control input *u*(*t*) are shown in Figs. 3 and 4, respectively. Then we increase the magnitude of $\alpha _1$ to a relatively large value, *i.e.*, $\alpha _1 = 5$ where the simulation results are shown in Figs. 5 and 6. One can see that, compared to the STA and PI methods, the proposed ESTA method has the fastest settling time and the smallest overshoot. In particular, for the moderate external disturbance with $\alpha _1 = 2$, the system settling time $t_s$ of the proposed ESTA is reduced from 10 and 4 seconds to 3.2 second ($68\%$ reduction and $20\%$ reduction) compared to those of the existing PI and STA methods, respectively. For the relatively large external disturbance $\alpha _1 = 5$, the $t_s$ of the proposed ESTA is reduced from 8.1 and 9.2 seconds to 6.1 second ($25\%$ reduction and $34\%$ reduction) respectively compared to those of the PI and STA methods (see $t_s$ in Table 1). Simultaneously, compared to the PI and STA methods the percentage of the system overshoot *O*.*S*. by using the proposed ESTA is respectively reduced from $30\%$ and $40\%$ to $25\%$ for the moderate external disturbance. Meanwhile, the *O*.*S*. by ESTA is reduced to $16\%$ for the relatively large external disturbance (see *O*.*S*. in Table 1). Note, the percentage of *O*.*S*. is calculated from the control signals (Figs. 3 and 5) because we choose the system steady state at its equilibrium state, *i.e.*, $\sigma (\infty ) = 0$.

**Table 1:** Settling times (obtained from Figs. 4 and 6) and overshoots (obtained from Figs. 3 and 5) by using three control methods for the nonlinear system (4) under constant external disturbances only, *i.e.*, $\alpha _2 = \alpha _3 = 0$.

$\alpha _1$	Settling time $t_s$ (Seconds)			Overshoot *O*.*S*. ($\%$)		
	PI	STA	ESTA	PI	STA	ESTA
2	10	4	**3.2**	30	40	**25**
5	8.1	9.2	**6.1**	30	40	**16**

Significant values are in [bold].

#### System response under external disturbances and linear unmodelled dynamics

To test the system response under various linear parameter uncertainties (or unmodelled dynamics), we keep the magnitude of the external disturbance unchanged, *i.e.*, $\alpha _1 = 2$, no higher order polynomial disturbance, *i.e.*, $\alpha _3 = 0$, and choose different values of linear unmodelled dynamics $\alpha _2$.

**Figure 7:**
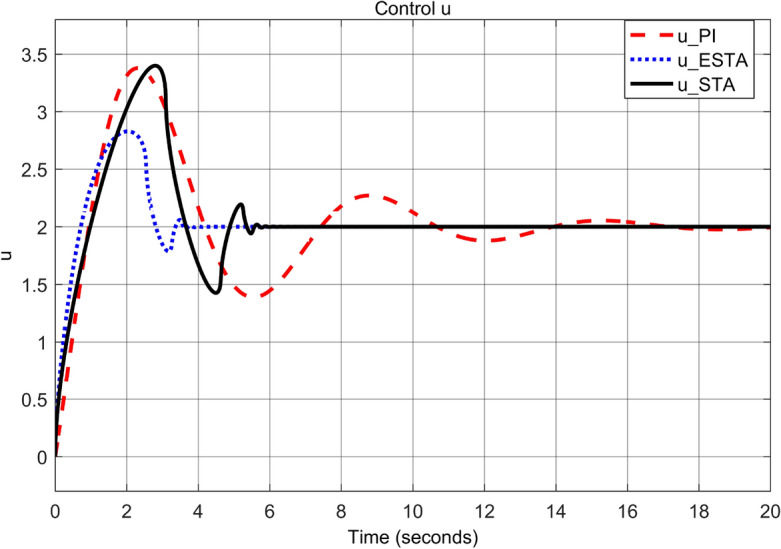
System response under external disturbances and linear unmodelled dynamics (35) with $\alpha _1 = 2$, $\alpha _2 = {\textbf {0.5}}$ and $\alpha _3 = 0$: Control inputs *u*(*t*) using PI (36) (dash red), ESTA (23) (dot blue), and STA (9) (solid black).

**Figure 8:**
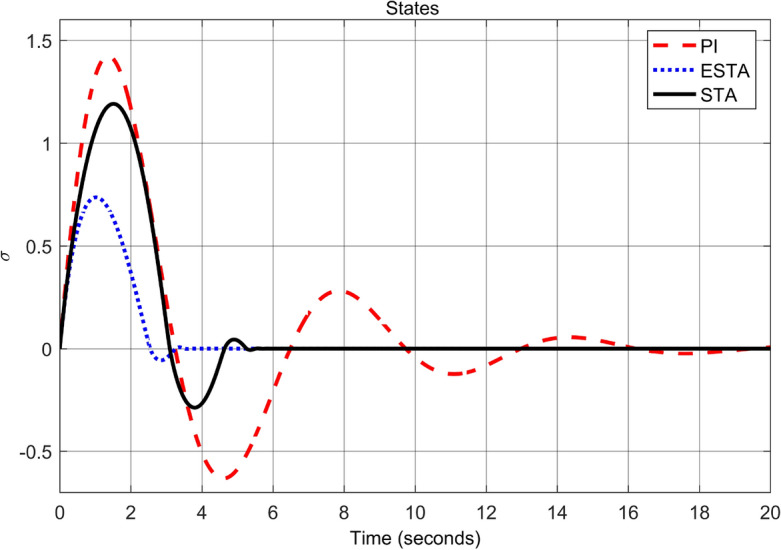
System response under external disturbances and linear unmodelled dynamics (35) with $\alpha _1 = 2$, $\alpha _2 = {\textbf {0.5}}$ and $\alpha _3 = 0$: States $\sigma (t)$ using PI (36) (dash red), ESTA (23) (dot blue), and STA (9) (solid black).

**Figure 9:**
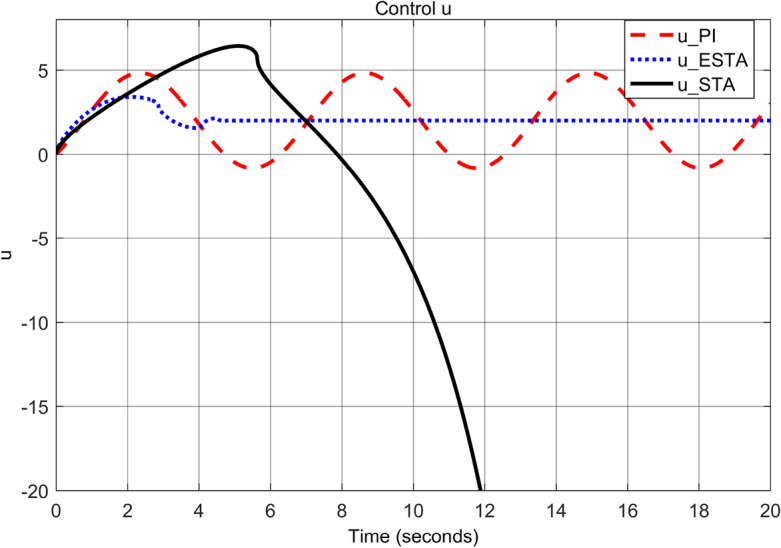
System response under external disturbances and linear unmodelled dynamics (35) with $\alpha _1 = 2$, $\alpha _2 = {\textbf {1.0}}$ and $\alpha _3 = 0$: Control inputs *u*(*t*) using PI (36) (dash red), ESTA (23) (dot blue), and STA (9) (solid black).

**Figure 10:**
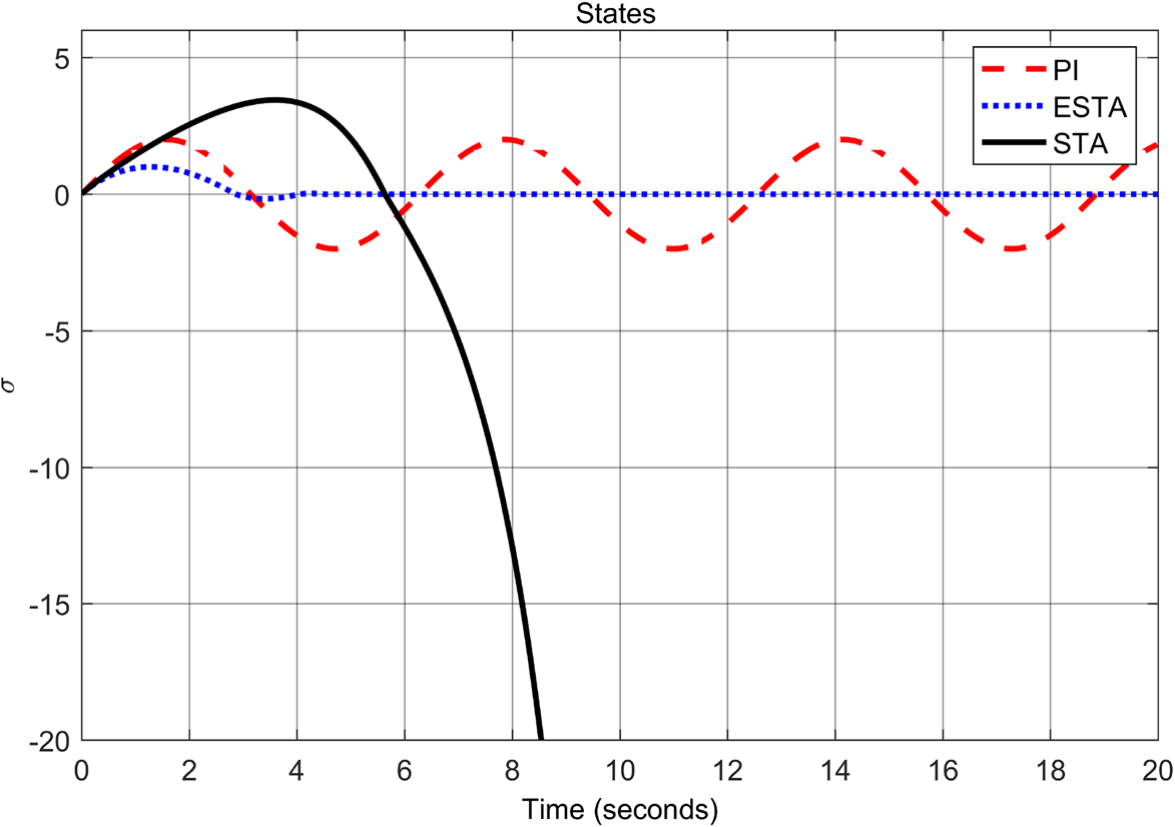
System response under external disturbances and linear unmodelled dynamics (35) with $\alpha _1 = 2$, $\alpha _2 = {\textbf {1.0}}$ and $\alpha _3 = 0$: States $\sigma (t)$ using PI (36) (dash red), ESTA (23) (dot blue), and STA (9) (solid black).

First we choose a relatively small value of $\alpha _2 = 0.5$. One can see that the ESTA method still has the fastest settling time and the smallest overshoot (see Figs. 7 and 8). Specifically, the settling time $t_s$ dropped from 18 and 5.2 seconds respectively by applying PI and STA approaches to 3.2 seconds by using the proposed ESTA method (see $t_s$ in Table 2). Then we increase the parameter uncertainty to a moderate level of $\alpha _2 = 1.0$. From the simulation results Figs. 9 and 10, one can see that by using STA and PI methods the systems are unstable and oscillating, respectively. In contrast, the system is still stable by using the proposed ESTA control method.

**Figure 11:**
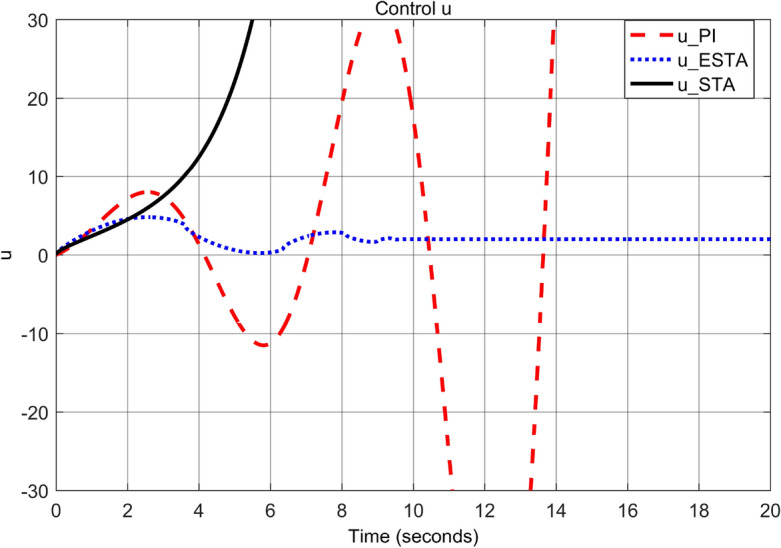
System response under external disturbances and linear unmodelled dynamics (35) with $\alpha _1 = 2$, $\alpha _2 = {\textbf {1.5}}$ and $\alpha _3 = 0$: Control inputs *u*(*t*) using PI (36) (dash red), ESTA (23) (dot blue), and STA (9) (solid black).

**Figure 12:**
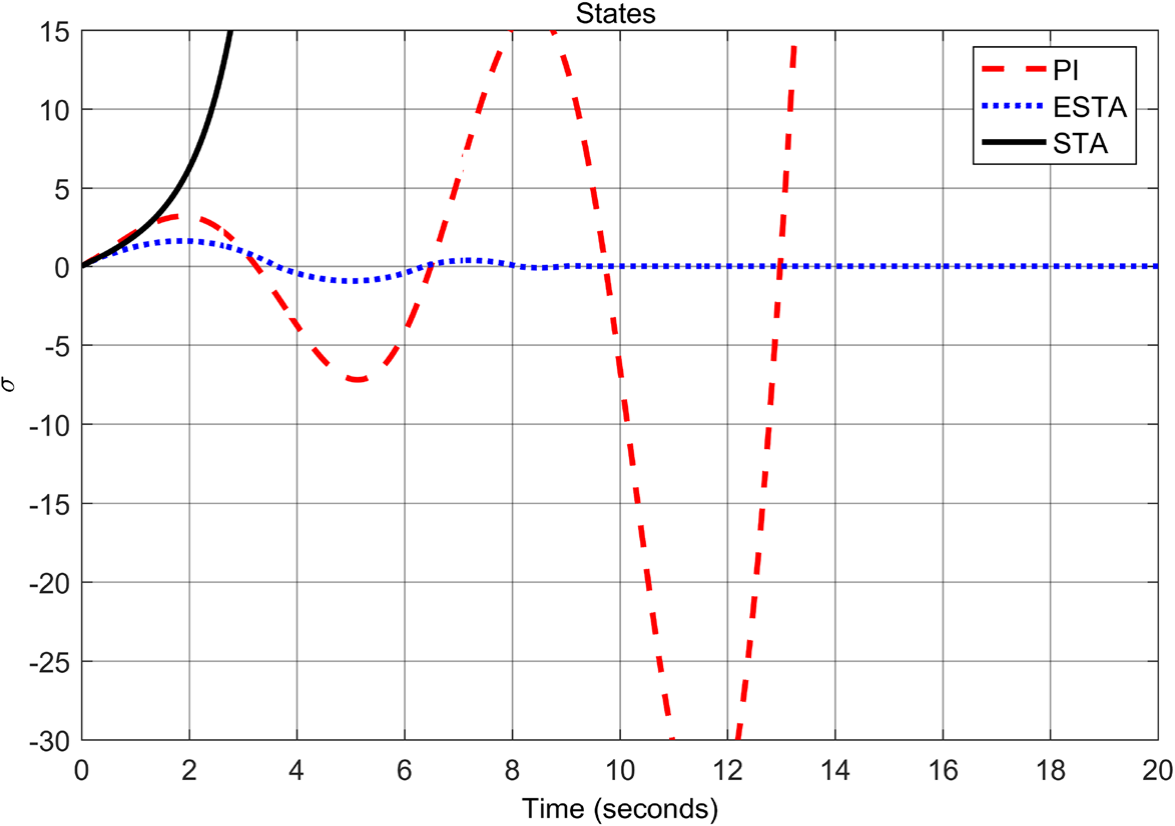
System response under external disturbances and linear unmodelled dynamics (35) with $\alpha _1 = 2$, $\alpha _2 = {\textbf {1.5}}$ and $\alpha _3 = 0$: States $\sigma (t)$ using PI (36) (dash red), ESTA (23) (dot blue), and STA (9) (solid black).

Note that any $\alpha _2 > d_1 = 1$ will lead to an unstable PI control as well. We continue to increase the $\alpha _2$ value to 1.5 where the simulation results are shown in Figs. 11 and 12. It can be seen that the system becomes unstable by using the PI method as well as by using the STA method. In contrast, the system state is still stable by applying the proposed ESTA method.

**Table 2:** Settling times (obtained from Fig. 8, 10 and 12) and overshoots (obtained from Figs. 7, 9, and 11) by using three control methods for the nonlinear system (4) under external disturbances and linear unmodelled dynamics, *i.e.*, $\alpha _1 = 2$, $\alpha _2 \ne 0$, and $\alpha _3 = 0$.

$\alpha _2$	Settling time $t_s$ (Seconds)			Overshoot *O*.*S*. ($\%$)		
	PI	STA	ESTA	PI	STA	ESTA
0.5	18	5.2	**3.2**	70	70	**40**
1.0	Oscillating	Unstable	**4.5**	Oscillating	Unstable	**50**
1.5	Unstable	Unstable	**9.5**	Unstable	Unstable	**150**

Significant values are in [bold].

#### System response under high order polynomial bounded disturbances

**Figure 13:**
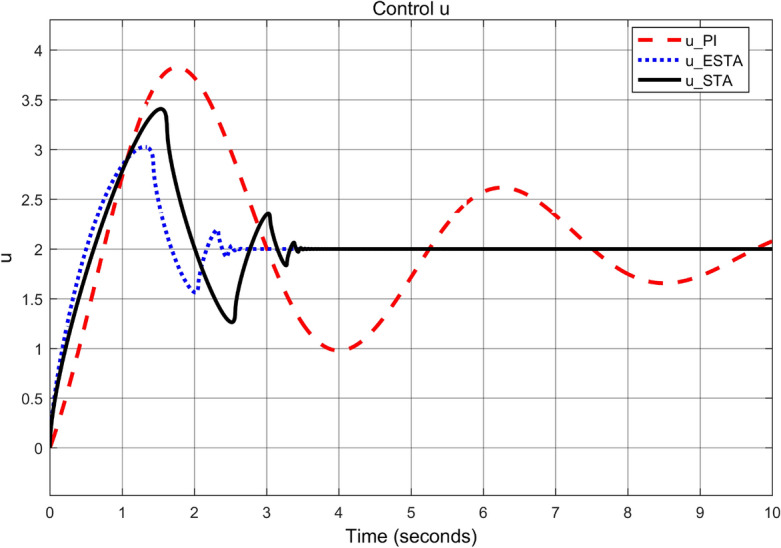
System response under parameter uncertainty (35) with $\alpha _1 = 2$, $\alpha _2 = 0.5$ and $\alpha _3 = {\textbf {0.1}}$: Control inputs *u*(*t*) using PI (36) (dash red), ESTA (23) (dot blue), and STA (9) (solid black). One can see that all the three methods are stable.

**Figure 14:**
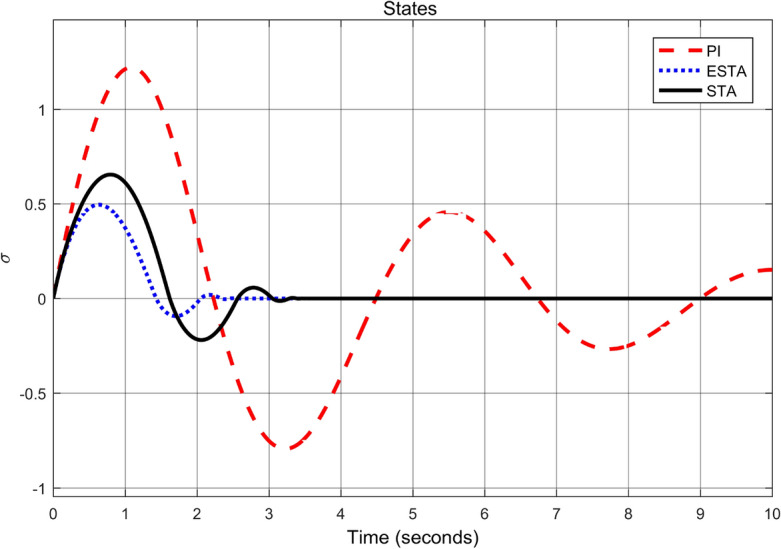
System response under parameter uncertainty (35) with $\alpha _1 = 2$, $\alpha _2 = 0.5$ and $\alpha _3 = {\textbf {0.1}}$: States $\sigma (t)$ using PI (36) (dash red), ESTA (23) (dot blue), and STA (9) (solid black). One can see that all the three methods are stable.

**Figure 15:**
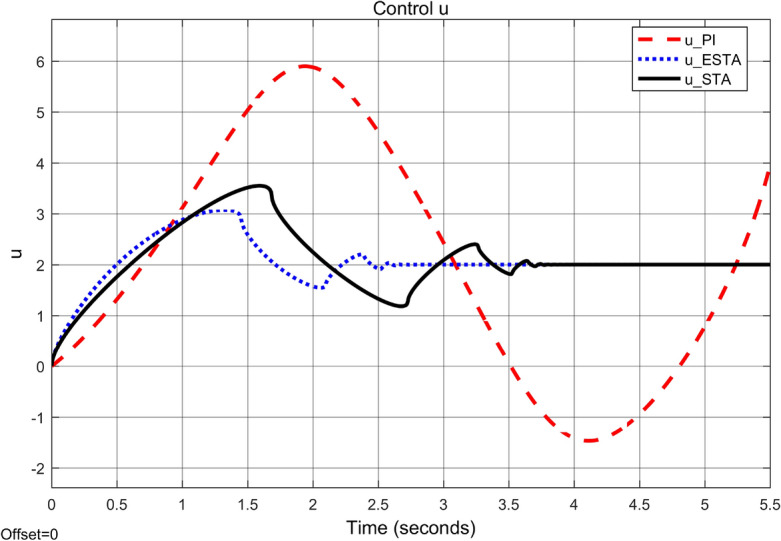
System response under parameter uncertainty (35) with $\alpha _1 = 2$, $\alpha _2 = 0.5$ and $\alpha _3 = {\textbf {0.25}}$: Control inputs *u*(*t*) using PI (36) (dash red), ESTA (23) (dot blue), and STA (9) (solid black). One can see that the PI method becomes unstable.

**Figure 16:**
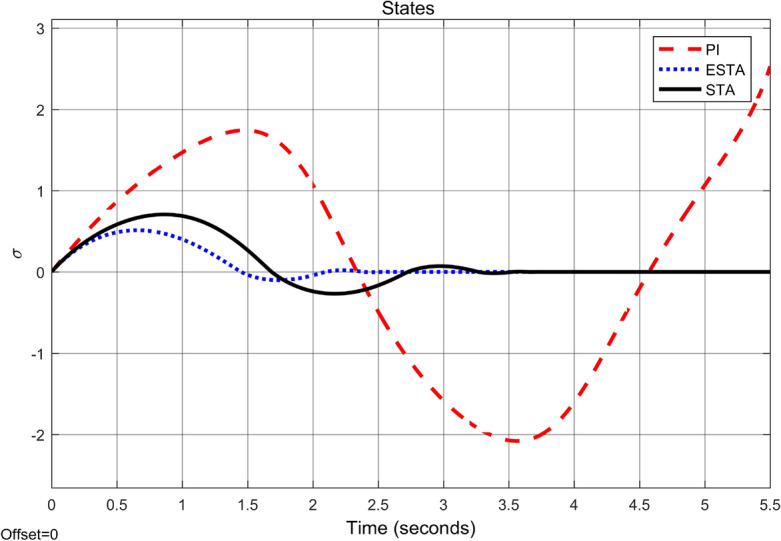
System response under parameter uncertainty (35) with $\alpha _1 = 2$, $\alpha _2 = 0.5$ and $\alpha _3 = {\textbf {0.25}}$: States $\sigma (t)$ using PI (36) (dash red), ESTA (23) (dot blue), and STA (9) (solid black). One can see that the PI method becomes unstable.

**Figure 17:**
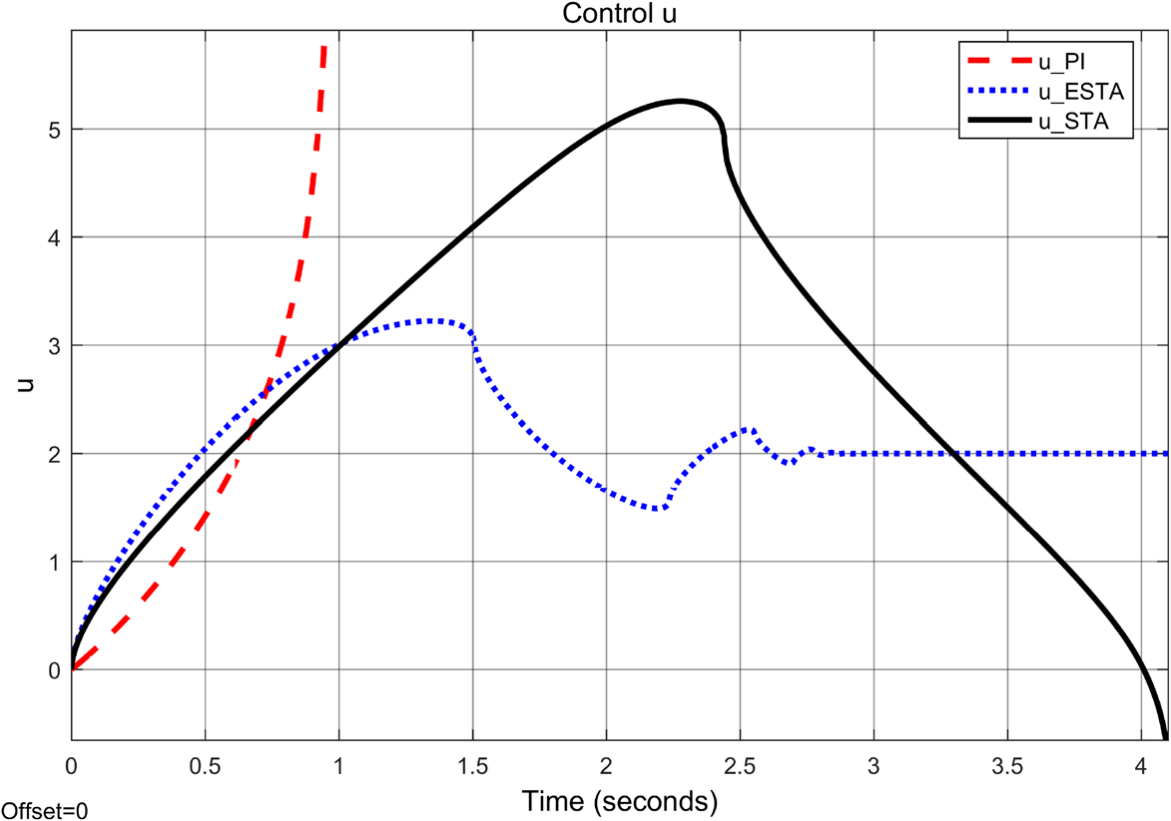
System response under parameter uncertainty (35) with $\alpha _1 = 2$, $\alpha _2 = 0.5$ and $\alpha _3 = {\textbf {0.57}}$: Control inputs *u*(*t*) using PI (36) (dash red), ESTA (23) (dot blue), and STA (9) (solid black). One can see that both PI and STA methods become unstable.

**Figure 18:**
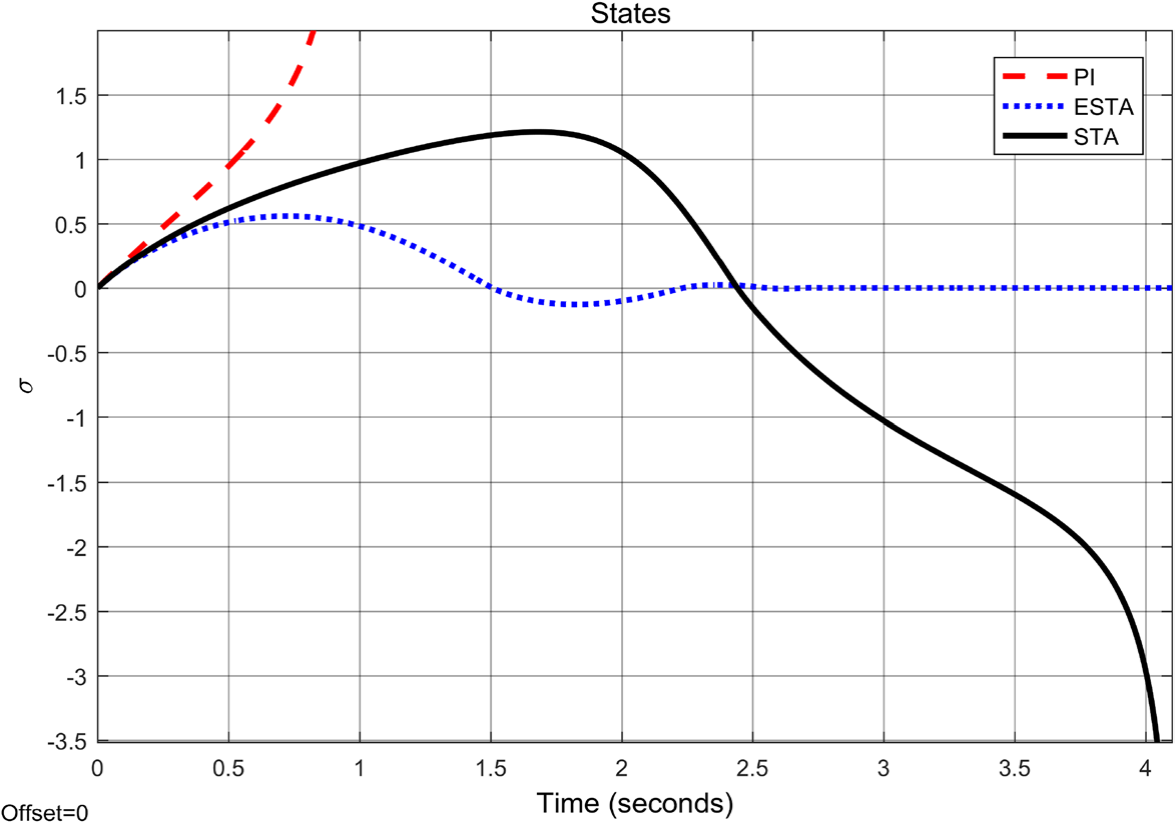
System response under parameter uncertainty (35) with $\alpha _1 = 2$, $\alpha _2 = 0.5$ and $\alpha _3 = {\textbf {0.57}}$: States $\sigma (t)$ using PI (36) (dash red), ESTA (23) (dot blue), and STA (9) (solid black). One can see that both PI and STA methods become unstable.

**Figure 19:**
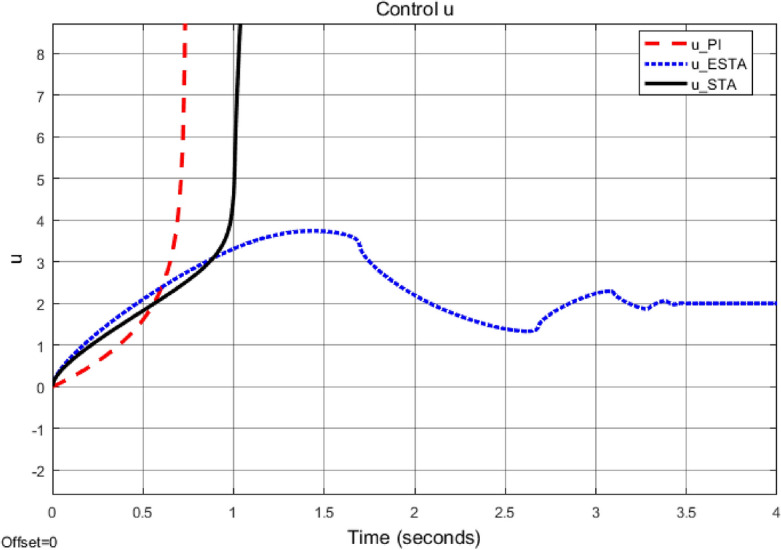
System response under parameter uncertainty (35) with $\alpha _1 = 2$, $\alpha _2 = 0.5$ and $\alpha _3 = {\textbf {1.0}}$: Control inputs *u*(*t*) using PI (36) (dash red), ESTA (23) (dot blue), and STA (9) (solid black). One can see that the ESTA method is still stable.

**Figure 20:**
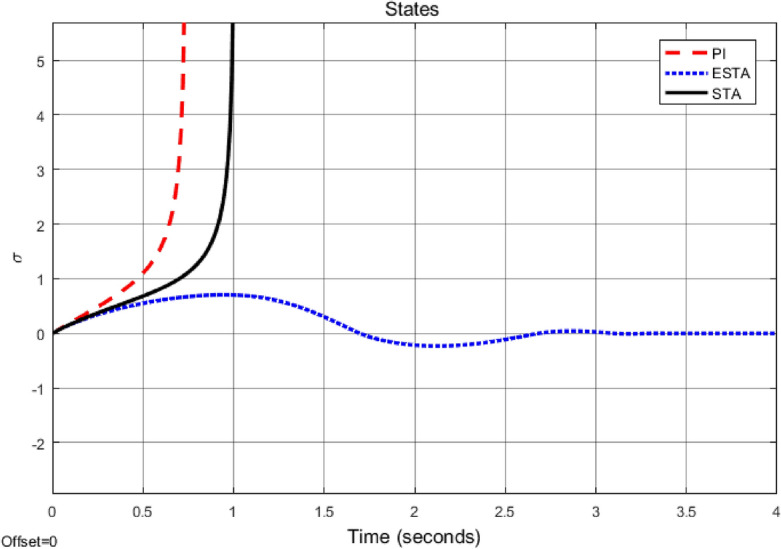
System response under parameter uncertainty (35) with $\alpha _1 = 2$, $\alpha _2 = 0.5$ and $\alpha _3 = {\textbf {1.0}}$: States $\sigma (t)$ using PI (36) (dash red), ESTA (23) (dot blue), and STA (9) (solid black). One can see that the ESTA method is still stable.

Now we test the system response where higher order polynomial bounded disturbances arise. We keep the magnitude of the external disturbance and linear unmodelled dynamics unchanged, *i.e.*, $\alpha _1 = 2$ and $\alpha _2 = 0.5$. We choose different values of $\alpha _3$ to see the system response when higher order polynomial disturbances arise. First we choose a relatively small value of $\alpha _3 = 0.1$. One can see that the ESTA method still has the fastest settling time and the smallest overshoot (see Figs. 13 and 14). Specifically, compared with PI and STA approaches, the settling time $t_s$ dropped from 15 and 3.1 seconds to 2.3 seconds and the overshoot *O*.*S*. reduced from $90\%$ and $70\%$ to $50\%$ by applying using the proposed ESTA method, respectively (see $t_s$ and *O*.*S*. in Table 3). Then we increase the magnitude of polynomial disturbance to a moderate level of $\alpha _3 = 0.25$. From the simulation results Figs. 15 and 16, one can see that by using the PI method the systems become unstable. When the magnitude of the polynomial disturbance coefficient is increased to $\alpha _3 = 0.57$, the STA method starts to become unstable as well (see Figs. 17 and 18). In contrast, the system keeps stable by using the proposed ESTA control method for the above three cases. Moreover, the system is still stable for ESTA as the coefficient value of polynomial disturbance arises to $\alpha _3 = 1$ (see Figs. 19 and 20).

**Table 3:** Settling times (obtained from Figs. 14, 16, 18, and 20) and overshoots (obtained from Figs. 13, 15, 17, and 19) by using three control methods for the nonlinear system (4) under higher order polynomial disturbances: $\alpha _1 = 2$ fixed, $\alpha _2 = 0.5$ fixed, and $\alpha _3 \ne 0$ varied.

$\alpha _3$	Settling time $t_s$ (Seconds)			Overshoot *O*.*S*. ($\%$)		
	PI	STA	ESTA	PI	STA	ESTA
0.1	15	3.1	**2.3**	90	70	**50**
0.25	Unstable	3.6	**2.5**	Unstable	75	**52**
0.57	Unstable	Unstable	**2.8**	Unstable	Unstable	**60**
1.0	Unstable	Unstable	**3.5**	Unstable	Unstable	**90**

Significant values are in [bold].

### Inverted pendulum

**Figure 21:**
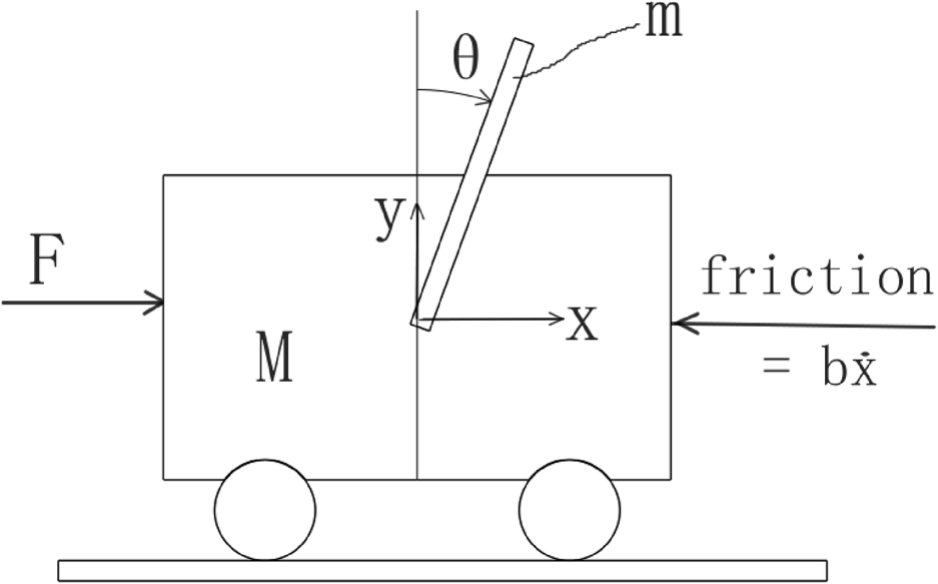
Schematic representation of the inverted pendulum system.

An inverted pendulum mounted to a motorized cart is commonly founded in control system textbooks and research literature^48^. In Fig. 21, *M* is the cart mass, *m* the pendulum mass, 2*l* the length of the pendulum, and *b* the coefficient of friction of the cart. In this case we only consider a two-dimensional problem where the pendulum is constrained to rotate in the vertical plane. The control input is the force *F* that moves the cart horizontally and the output is the angular position $\theta$ for simplicity. Since the angular velocity of the pendulum can be estimated via a filtered differentiator of the angular position $\theta$, a PID rather than a PI controller is often used in most literature to stabilize the pendulum system^48^. For the comparison, we stabilize the system using ESTA, PID and STA methods.

#### Nonlinear equations of motion

Applying Newton or energy method, we can derive the following nonlinear equations of motion.

37 $$\begin{aligned} (M + m) \ddot{x} + b {\dot{x}} + (m l \cos \theta ) \ddot{\theta } - (m l \sin \theta ) {\dot{\theta }}^2&= F \end{aligned}$$

38 $$\begin{aligned} (m l \cos \theta )\ddot{x} + (I + m l^2)\ddot{\theta } - g m l \sin \theta&= 0 \end{aligned}$$

where $I = (1/12) m (2l)^2 = \dfrac{1}{3}ml^2$ is the mass moment of inertia of the pendulum around the center of the pendulum and *g* is the gravity. Then we can solve the above equations (37) and (38) to obtain the following full nonlinear equation.

39 $$\begin{aligned} \ddot{\theta }&= \Big (b \cos \theta {\dot{x}} - (ml \sin \theta \cos \theta ) {\dot{\theta }}^2 + (M+m)g\sin \theta - (\cos \theta ) F \Big )/q \end{aligned}$$

40 $$\begin{aligned} \ddot{x}&= \Big (- \dfrac{4}{3}lb {\dot{x}} + \dfrac{4}{3}ml^2 \sin \theta {\dot{\theta }}^2 - gml\sin \theta \cos \theta + \dfrac{4}{3} l F \Big )/q \end{aligned}$$

where $q = \dfrac{4}{3}l(M+m) - ml\cos ^2 \theta > 0$. Let $\sigma _1 = k \theta + {\dot{\theta }}$ be a sliding variable^10^ and $u = - F$ be the new control input. The second-order differential equation (39) can then be converted into a first-order differential equation.

41 $$\begin{aligned} \dot{\sigma _1} = f(\theta , {\dot{\theta }}, x, {\dot{x}}) + g(\theta , {\dot{\theta }}, x, {\dot{x}})u \end{aligned}$$

where $f(\theta , {\dot{\theta }}, x, {\dot{x}}) = k {\dot{\theta }} + \Big (b \cos \theta {\dot{x}} - (ml \sin \theta \cos \theta ) {\dot{\theta }}^2 + (M+m)g\sin \theta \Big )/q$ and $g(\theta , {\dot{\theta }}, x, {\dot{x}}) = (\cos \theta )/q$. Note, $f(\theta , {\dot{\theta }}, x, {\dot{x}})$ is considered here to be a disturbance bounded by a quadratic polynomial, *i.e.*, all the coefficients $\dfrac{(M+m)g\sin \theta }{q}$, $[k, \dfrac{b \cos \theta }{q}]$, and $\dfrac{ml \sin \theta \cos \theta }{q}$ of the polynomial terms $\theta ^0$, $[{\dot{\theta }}^1, {\dot{x}}^1]^T$ and ${\dot{\theta }}^2$, respectively, are bounded. Thus, the polynomial bounded disturbance $f(\theta , {\dot{\theta }}, x, {\dot{x}})$ satisfies Assumption 1. Moreover, $g(\theta , {\dot{\theta }}, x, {\dot{x}}) > 0$ is positive definite for $-\pi /2< \theta < \pi /2$ and satisfies Assumption 2. Since the output is one measurement $\theta$ and the PID control law contains the differentiation of $\theta$, we use $\dot{\hat{\theta }}(t)$ as the filtered differentiation of $\theta$ to replace its mathematical differentiation ${\dot{\theta }}$, *i.e.*,

42 $$\begin{aligned} \dot{\hat{\theta }}(t) = {\mathscr {L}}^{-1} \{\dfrac{s}{T s + 1} \theta (s)\} \end{aligned}$$

where ${\mathscr {L}}^{-1}$ denotes the Laplace inverse transform. The PID feedback control law can then be written as

43 $$\begin{aligned} u_{PID}(\theta , \dot{\hat{\theta }}) = - d_1 \theta - d_2 \int _0^t \theta (\tau ) d \tau - d_3 \dot{\hat{\theta }}(t) \end{aligned}$$

For comparison, we use ESTA control law (34) and expand the structure of the control law STA (9) to be similar with the structure of PID (43).

44 $$\begin{aligned} u_{STA}(\theta , \dot{\hat{\theta }})&= - k_1 \sqrt{|\theta (t)|}\textrm{sgn}(\theta (t)) - k_2 \int _0^t \textrm{sgn}(\theta (\tau )) d \tau - k_3 \dot{\hat{\theta }}(t) \end{aligned}$$

#### Stabilization of the inverted pendulum

Now we can build the simulation model of the inverse pendulum system using the equations of motion (39) and (40) and applying the three control laws (34), (43) and (44) for the three methods ESTA, PID, and STA, respectively. The parameter values for the inverted pendulum system are shown in Table 4.

**Table 4:** Parameter values for the inverted pendulum mounted to a cart.

Parameter	Symbol	Value	Unit
Cart mass	*M*	1	Kg
Pendulum mass	*m*	0.1	Kg
Pendulum length	2*l*	1	m
Coefficient of friction for cart	*b*	0.1	N/(m/s)
Gravity	*g*	9.8	${\rm m/s}^2$
Pulse offset angle	$A_o$	0.1, 0.2, 0.4, 0.8, 1.2, 1.4	rad

**Figure 22:**
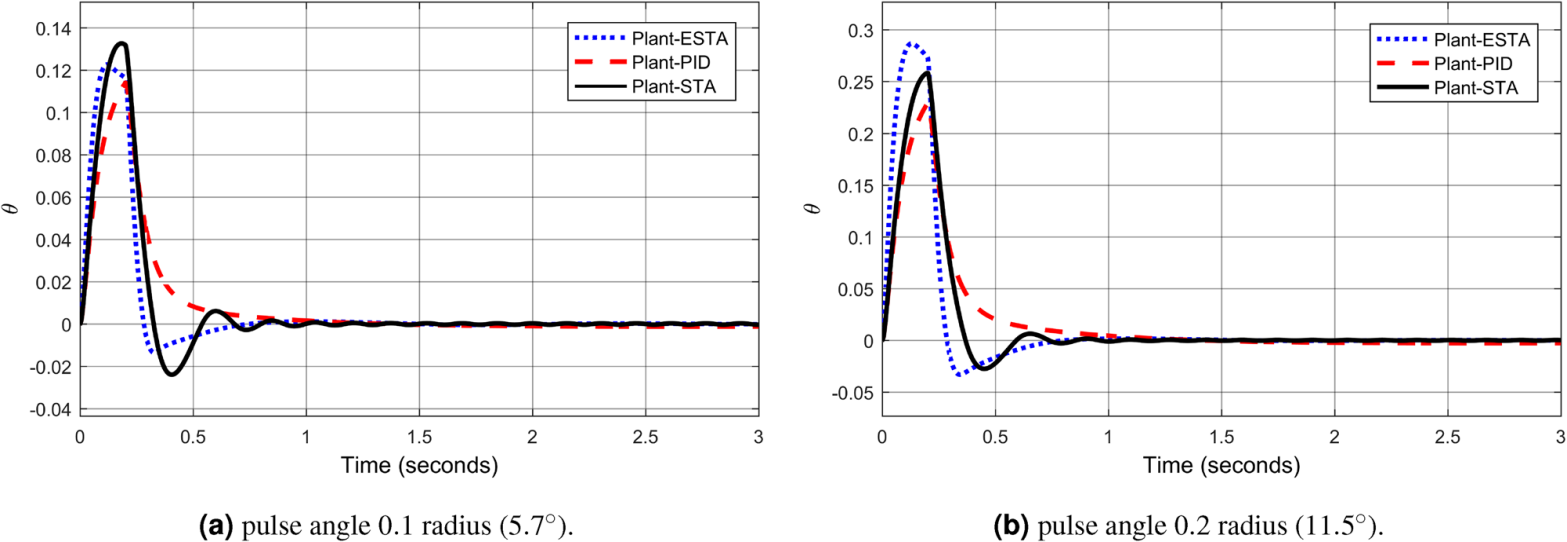
System responses under pulse disturbances with relatively small offset angles 0.1 radius ($5.7^\circ$) and 0.2 radius ($11.5^\circ$) using ESTA (23) (dot blue), PID (36) (dash red), and STA (9) (solid black).

**Figure 23:**
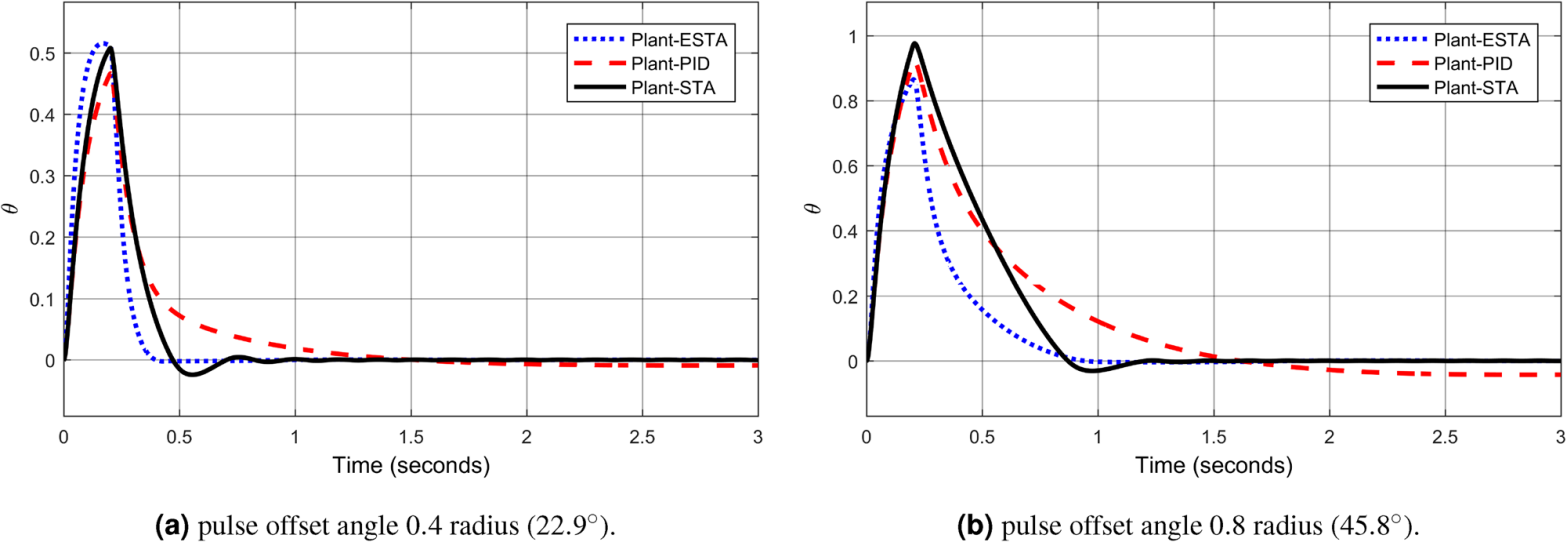
System responses under pulse disturbances with medium offset angles 0.4 radius ($22.9^\circ$) and 0.8 radius ($45.8^\circ$) using ESTA (23) (dot blue), PID (36) (dash red), and STA (9) (solid black).

**Figure 24:**
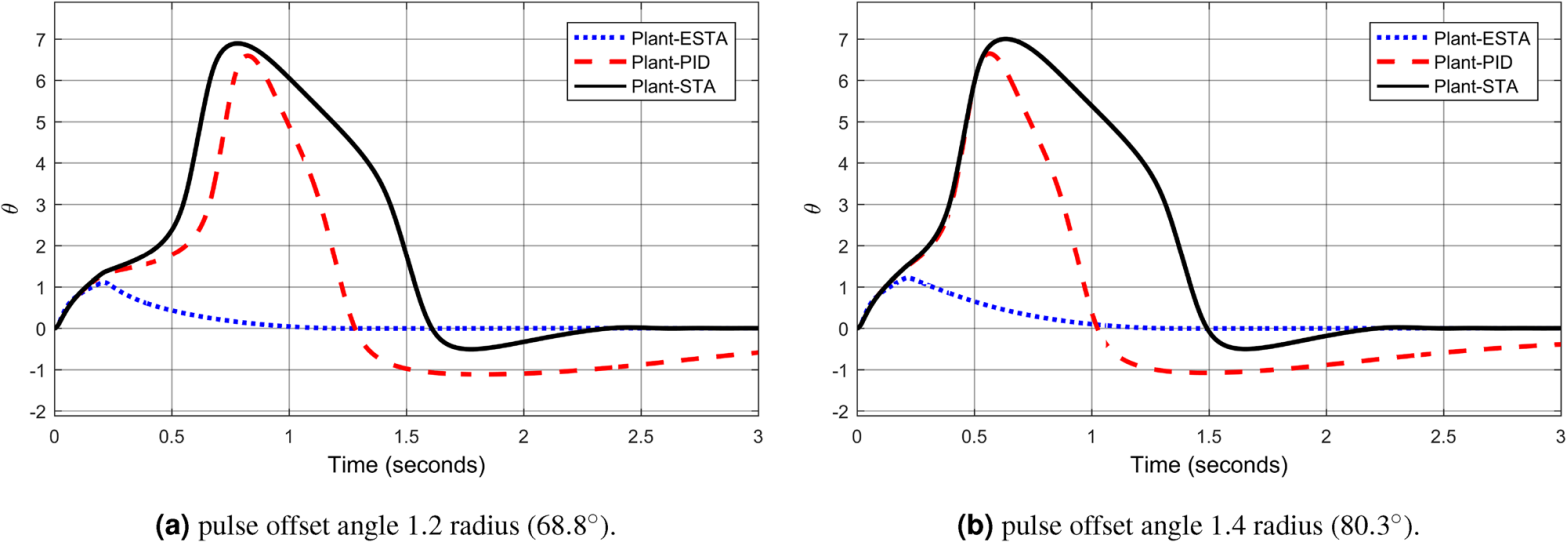
System responses under pulse disturbances with relatively large offset angles 1.2 radius ($68.8^\circ$) and 1.4 radius ($80.3^\circ$) using ESTA (23) (dot blue), PID (36) (dash red), and STA (9) (solid black).

We test the transient response of the inverted pendulum system under different pulse disturbances. First we tuned the PID control, and, after some try and error, it is found that the PID gains $[k_1, k_2, k_3] = [50, 10, 20]$ provides a satisfactory response. For comparison, let’s make the corresponding gains the same for all three methods, *i.e.*, $k_1 = c_1 = d_1 = 50$, $k_2 = c_2 = d_2 = 10$ and $k_3 = c_3 = d_3 = 20$. Let’s choose the pulse width 0.2 seconds fixed and select different pulse amplitudes $A_o$, 0.1, 0.2, 0.4, 0.8, 1.2 and 1.4 in radius, which corresponds to the magnitudes of the offset angles of the inverted pendulum, $5.7^\circ$, $11.5^\circ$, $22.9^\circ$, $45.8^\circ$, $68.8^\circ$ and $80.3^\circ$, respectively. The simulation results are shown in Figs. 22, 23 and 24. From Fig. 22 where the pulse offset angles $A_o$ are relatively small ($5.7^\circ$ and $11.5^\circ$), one can see that there is no significant difference in the overshoot and settling time of the system between the three different control methods. When the pulse angle offset $A_o$ increases to medium values, $22.9^\circ$ and $45.8^\circ$, and relatively large values, $68.8^\circ$ and $80.3^\circ$, the overshoot and settling time of the system are significantly different between the three methods (Figs. 23 and 24). In particular, for the pulse angle offset $A_o$ of medium values $22.9^\circ$ and $45.8^\circ$, the system settling time $t_s$ of the proposed ESTA is reduced from 3 and 0.8 seconds to 0.4 second ($83\%$ reduction and $50\%$ reduction) and 5 and 1.2 seconds to 0.9 second ($81\%$ reduction and $25\%$ reduction) compared to those of the existing PI and STA methods, respectively (see $t_s$ and *O*.*S* in Table 5). For $A_o$ of large values $68.8^\circ$ and $80.3^\circ$, one can see that by using STA and PI methods the systems are unstable. In contrast, the system is still stable by using the proposed ESTA control method (Fig. 24). Note that an offset angle $A_{o} \ge \dfrac{\pi }{2}=1.57$ radius ($90^\circ$) means that the inverted pendulum would fall down completely. In other words, $\theta \in (-\dfrac{\pi }{2}, \dfrac{\pi }{2})$ can be a possibly stabilizable range of the inverted pendulum. The performance of the inverted pendulum stabilized via the three methods is summarized in Table 5.

**Table 5:** Settling times and overshoots obtained from Figs. 22, 23 and 24 by using three control methods for the inverted pendulum system, (39) and (40), under external pulse disturbances with pulse width 0.2 seconds.

Pulse Amplitude $A_{o}$ ($rad/^\circ$)	Settling time $t_s$ (Seconds)			Overshoot *O*.*S*. ($\%$)		
	PI	STA	ESTA	PI	STA	ESTA
0.1/$5.7^\circ$	1.0	1.1	**0.9**	18	32	**24**
0.2/$11.5^\circ$	1.0	1.0	**0.9**	15	30	**40**
0.4/$22.9^\circ$	3	0.8	**0.4**	20	26	**28**
0.8/$45.8^\circ$	5	1.2	**0.9**	10	20	**9**
1.2/$68.8^\circ$	Unstable	Unstable	**1.0**	Unstable	Unstable	**5**
1.4/$80.3^\circ$	Unstable	Unstable	**2.8**	Unstable	Unstable	**5**

Significant values are in [bold].

#### Phase portraits of angular positions and angular velocities

**Figure 25:**
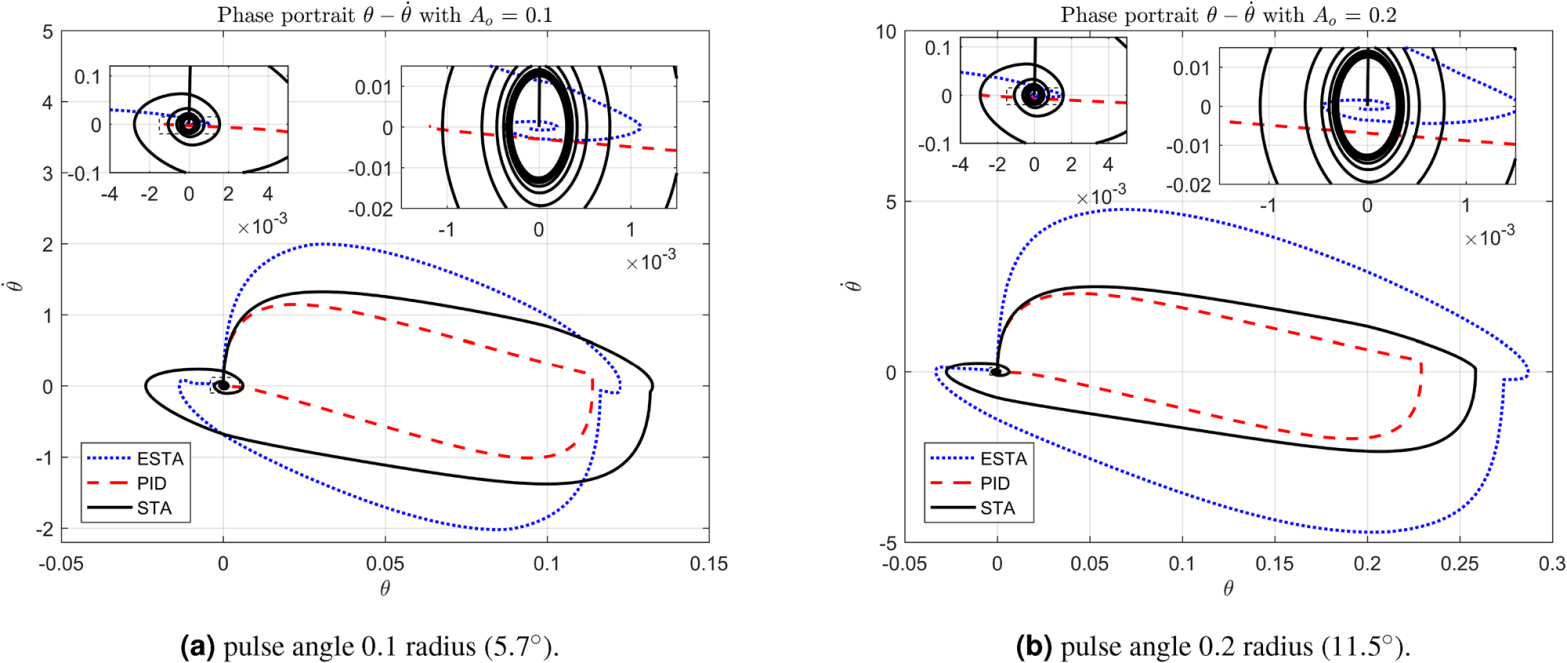
Phase portraits of angular positions and velocities under pulse disturbances with relatively small offset angles 0.1 radius ($5.7^\circ$) and 0.2 radius ($11.5^\circ$) using ESTA (23) (dot blue), PID (36) (dash red), and STA (9) (solid black).

**Figure 26:**
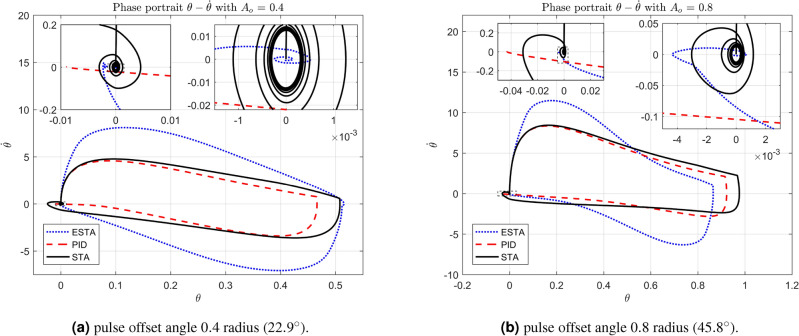
Phase portraits of angular positions and velocities under pulse disturbances with medium offset angles 0.4 radius ($22.9^\circ$) and 0.8 radius ($45.8^\circ$) using ESTA (23) (dot blue), PID (36) (dash red), and STA (9) (solid black).

**Figure 27:**
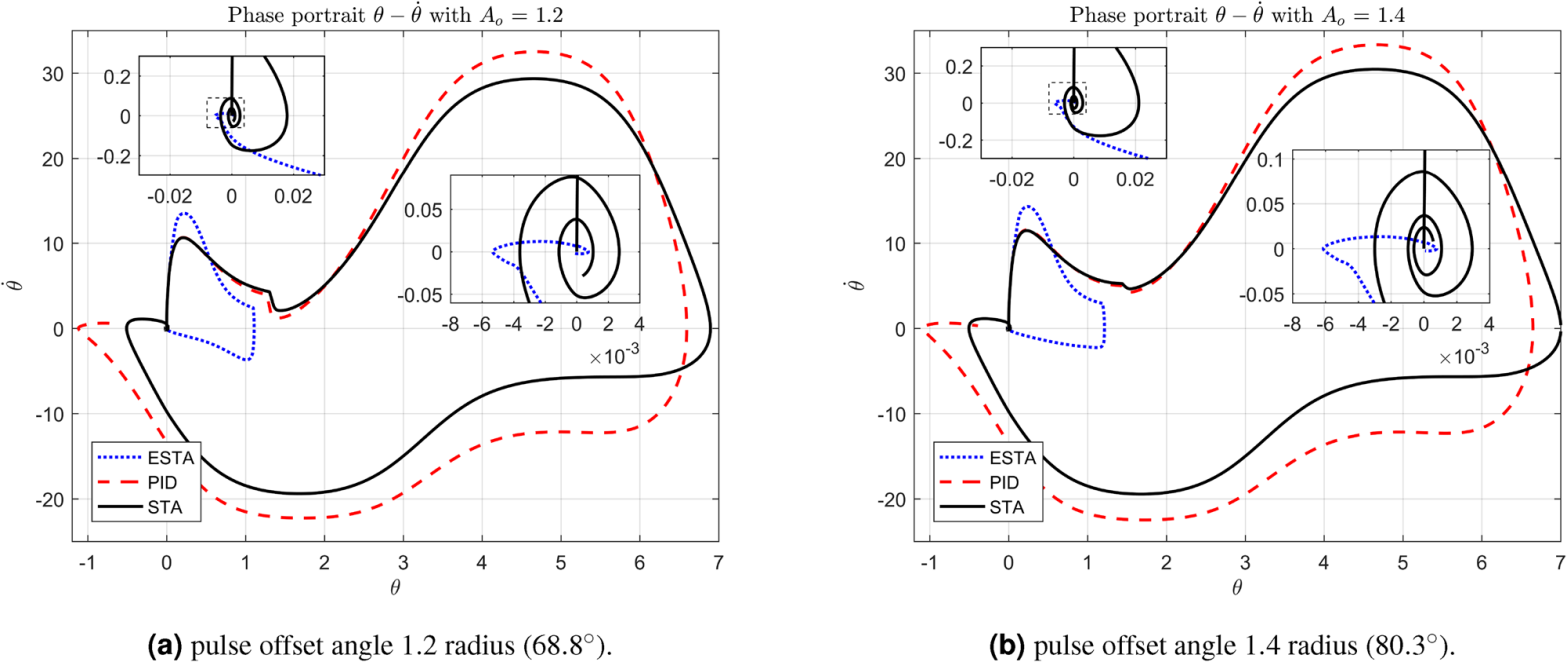
Phase portraits of angular positions and velocities under pulse disturbances with relatively large offset angles 1.2 radius ($68.8^\circ$) and 1.4 radius ($80.3^\circ$) using ESTA (23) (dot blue), PID (36) (dash red), and STA (9) (solid black).

The simulation results of the inverted pendulum system also let us analyse the phase portraits of the system. In the phase portraits Figs. 25-27 the angular position state $\theta$ versus the angular velocity state ${\dot{\theta }}$ are plotted along with two levels of local magnifications. For the system under pulse disturbances with relatively small offset angles ($A_o = 0.1$ and $A_o = 0.2$, Fig. 25) and medium offset angles ($A_o = 0.4$ and $A_o = 0.8$, Fig. 26), one can see that the trajectories of the two phases $\theta$ and ${\dot{\theta }}$ have similar performance with the three methods. All $\theta - {\dot{\theta }}$ trajectories start vertically upwards from the origin and eventually return in a spiral towards the origin. Compared to the STA and PID methods, the trajectory ranges of the ESTA method are slightly larger, but the returns are much closer to the origin. For the offset angles increased to large values ($A_o = 1.2$ and $A_o = 1.4$), the proposed ESTA method performed much better than the STA and PI methods (Fig. 27). Specifically, not only the $\theta - {\dot{\theta }}$ trajectory regression to the origin of the ESTA method is faster and closer, but also the trajectory range is much smaller.

## Conclusion

The paper first investigates some perturbation assumptions in some STA designs over the past decade. The polynomial perturbation is then used to fix the stability issue and further extend the range of nonlinear dynamic systems. To handle the extended systems, a novel adaptive super-twisting sliding mode control with exponential reaching law is proposed. The new ESTA method is extended from the existing STA design and integrated with an novel exponential reaching law. The stability analysis and finite-time convergence are proven using Lyapunov theory and an intuitive analysis of the control behavior. The new design successfully applied to the control of the nonlinear systems having lumped perturbations bounded by a parameter-unknown-a-priori polynomial. The simulation results of the illustrated example show that, for the external disturbance, the system settling time $t_s$ of the proposed ESTA is reduced by $25-68\%$ and $20-35\%$ compared to those of the existing PID and STA methods, respectively. Simultaneously, compared to the PI and STA methods the percentage of the system overshoot *O*.*S*. by using the proposed ESTA is respectively reduced from $30\%$ and $40\%$ to $16-25\%$. It is worth noting that when the polynomial disturbance appears and arises to medium or relatively large amplitudes, the system using STA and PI methods began to become unstable, but with the proposed ESTA control method it remains stable. The simulation results of the inverted pendulum also show that, when the inverted pendulum is disturbed by small pulses close to the equilibrium point, the use of all three methods has a good stabilizing effect. However, when the pulse is of medium size, ESTA has better stability than PID and STA. Specifically, ESTA method reduced settling time by $25-80\%$ and overshoot by $10-50\%$. Moreover, when pulse is large, ESTA can still stabilize the system quickly, while PID and STA cannot stabilize the system.

Compared with the commonly used STA and PID methods, the proposed ESTA approaches exhibit superiority in terms of faster settling time, smaller overshoot and stronger stability. Moreover, ESTA methods are able to deal with nonlinear systems where higher order polynomial bounded disturbances may arise. Thus, the proposed ESTA methods are of great significance for future designs and applications of robust control for a large class of nonlinear dynamic systems, such as all-weather flight control of civil airliners, flight control of rescue helicopters in disaster weather, motion control of humanoid robots subjected to sudden external forces, *etc.*. Our future work will focus on a real flight control of an unmanned aerial vehicle in strong wind environments.

## Data Availability

The datasets generated during and/or analysed during the current study are available from the corresponding author upon reasonable request.

## Appendicies

### Proof of the Lemma

#### Proof

There are two cases of $|\sigma | \ne 0$.

- Case I: $\sigma > 0$. Then, $\textrm{sgn}(\sigma (t)) = 1$. From (2), we have
  45 $$\begin{aligned} \frac{d}{d t} |\sigma (t)| = {\dot{\sigma }} (t) = {\dot{\sigma }} (t) \textrm{sgn}(\sigma (t)) \end{aligned}$$
- Case II: $\sigma < 0$. Then, $\textrm{sgn}(\sigma (t)) = -1$.
  46 $$\begin{aligned} \frac{d}{d t} |\sigma (t)| = - {\dot{\sigma }} (t) = {\dot{\sigma }} (t) \textrm{sgn}(\sigma (t)) \end{aligned}$$

$$\square $$

Note, the above lemma does not include the case of $\sigma =0$ where the system is under the steady state.

### Proof of the Proposition 1

#### Proof

Consider the case $|\sigma | \ge \varepsilon > 0$. Using the definition of $h(|\sigma |)$ (Eq. (30)), we have from (5)

47 $$\begin{aligned} f&(\sigma , t)\textrm{sgn}(\sigma ) - g(\sigma , t) c_1 \dfrac{|\sigma |}{|\sigma | + \mu } h(|\sigma |) \nonumber \\&\le |f(\sigma , t)| - {\underline{b}} c_1 \dfrac{\varepsilon }{\varepsilon + \mu } h(|\sigma |) \nonumber \\&\le {\underline{b}} c_1 \dfrac{\varepsilon }{\varepsilon + \mu } \left( 1 + \sqrt{|\sigma |} \right) + \sum ^{q}_{i = 0} a_i | \sigma |^i - {\underline{b}} c_1 \dfrac{\varepsilon }{\varepsilon + \mu } e^{\sqrt{|\sigma |}} \nonumber \\&\le c^* \end{aligned}$$

for a positive constant $c^*$. Now we consider the time derivative of $|\sigma |$. Using Lemma 1 and (47), we obtain for $|\sigma | \ge \varepsilon > 0$,

$$\begin{aligned} \frac{d}{d t} |\sigma |&= f(\sigma , t) \textrm{sgn}(\sigma ) + g(\sigma , t) \textrm{sgn}(\sigma ) u \\&= f(\sigma , t) \textrm{sgn}(\sigma ) - g(\sigma , t) \textrm{sgn}(\sigma ) c_1 \left( e^{ \sqrt{|\sigma |}} - 1 \right) \dfrac{\sigma }{|\sigma | + \mu } \\&\hspace{4mm} - g(\sigma , t)\textrm{sgn}(\sigma ) c_2 \int _0^t \sigma (\tau ) d \tau \\&= f(\sigma , t) \textrm{sgn}(\sigma ) - g(\sigma , t) \dfrac{|\sigma |}{|\sigma | + \mu } c_1 \left( \sqrt{|\sigma |} + h(|\sigma |) \right) \\&\hspace{4mm} - c_2g(\sigma , t)\textrm{sgn}(\sigma ) \int _0^t \sigma (\tau ) d \tau \\&\le \left( c^* - c_2 {\underline{b}} \int _0^t |\sigma (\tau )| d \tau \right) - c_1 {\underline{b}} \dfrac{\varepsilon }{\varepsilon + \mu } \sqrt{|\sigma |} \end{aligned}$$

Noting that $\int _0^t |\sigma (\tau )| d \tau$ is continuously increasing whenever $|\sigma | \ge \varepsilon > 0$, there exists a time $t^*$ such that $\forall t \ge t^*$, $\left( c^* - c_2 {\underline{b}} \int _0^t |\sigma (\tau )| d \tau \right) \le 0$. Then, after time $t^*$ ($t \ge t^*$),

$$\begin{aligned} \frac{d}{d t} |\sigma | \le - c_1 {\underline{b}} \dfrac{\varepsilon }{\varepsilon + \mu } \sqrt{|\sigma |} \end{aligned}$$

Similar to the proof of Theorem 1, we conclude that $|\sigma |$ reaches the region $|\sigma | \le \varepsilon$ in finite time with the reaching time estimated as $t_F \le \dfrac{2(\varepsilon + \mu )}{c_1 {\underline{b}} \varepsilon } \sqrt{|\sigma (t^*)|} + t^*$ $\square$

## References

[1] Gao W, Hung J-C (1993). Variable structure control of nonlinear systems: A new approach. IEEE Trans. Industr. Electron..

[2] Utkin VI (1992). Sliding Modes in Control and Optimization.

[3] Khalil H (2002). Nonlinear Systems.

[4] Shtessel YB, Shkolnilov IA, Brown MDJ (2003). An asymptotic second-order smooth sliding mode control. Asian J. Control.

[5] Bartolini G, Fridman L, Pisano A, Usai E (2008). Modern Slidng Mode Control Theory—New Perspectives and Aplications.

[6] Taleb M, Plestan F, Bououlid B (2015). An adaptive solution for robust control based on integral high-order sliding mode concept. Int. J. Robust Nonlinear Control.

[7] Levant A (2001). Universal single-input-single-output (SISO) sliding-mode controllers with finite-time convergence. IEEE Trans. Autom. Control.

[8] Ding S, Park JH, Chen C-C (2020). Second-order sliding mode controller design with output constraint. Automatica.

[9] Levant A (1993). Sliding order and sliding accuracy in sliding mode control. Int. J. Control.

[10] Utkin VI (2013). On convergence time and disturbance rejection of super-twisting control. IEEE Trans. Autom. Control.

[11] Chalanga A, Kamal S, Fridman LM, Bandyopadhyay B, Moreno JA (2016). Implementation of super-twisting control: Super-twisting and higher order sliding-mode observer-based approaches. IEEE Trans. Industr. Electron..

[12] Seeber R, Horn M, Fridman L (2018). A novel method to estimate the reaching time of the super-twisting algorithm. IEEE Trans. Autom. Control.

[13] Colotti A, Monnet D, Goldsztejn A, Plestan F (2022). New convergence conditions for the super twisting algorithm with uncertain input gain. Automatica.

[14] Nurettin A, İnanç N (2023). Sensorless vector control for induction motor drive at very low and zero speeds based on an adaptive-gain super-twisting sliding mode observer. IEEE J. Emerg. Sel. Top. Power Electron..

[15] Perez-Ventura U, Fridman LM (2019). Design of super-twisting control gains: A describing function based methodology. Automatica.

[16] Yan Y, Yu S, Yu X (2019). Quantized super-twisting algorithm based sliding mode control. Automatica.

[17] Polyakov A, Poznyak A (2009). Reaching time estimation for super-twisting second order sliding mode controller via lyapunov function designing. IEEE Trans. Autom. Control.

[18] Seeber R, Reichhartinger M (2020). Conditioned Super-Twisting Algorithm for systems with saturated control action. Automatica.

[19] Papageorgiou D, Edwards C (2022). On the behaviour of under-tuned super-twisting sliding mode control loops. Automatica.

[20] Zhao Z, Yang J, Li S, Zhang Z, Guo L (2015). Finite-time super-twisting sliding mode control for Mars entry trajectory tracking. J. Franklin Inst..

[21] Tayebi-Haghighi S, Piltan F, Kim JM (2018). Robust composite high-order super-twisting sliding mode control of robot manipulators. Robotics.

[22] Kali Y, Saad M, Benjelloun K, Khairallah C (2018). Super-twisting algorithm with time delay estimation for uncertain robot manipulators. Nonlinear Dyn..

[23] Matraji I, Al-Wahedi K, Al-Durra A (2020). Higher-order super-twisting control for trajectory tracking control of skid-steered mobile robot. IEEE Access.

[24] Gonzalez-Hernandez I, Palacios FM, Cruz SS, Quesada ESE, Leal RL (2017). Real-time altitude control for a quadrotor helicopter using a super-twisting controller based on high-order sliding mode observer. Int. J. Adv. Rob. Syst..

[25] Zhang C, Chen T, Shang W, Zheng Z, Yuan H (2021). Adaptive super-twisting distributed formation control of multi-quadrotor under external disturbance. IEEE Access.

[26] Liu J, Sun M, Chen Z, Sun Q (2020). Super-twisting sliding mode control for aircraft at high angle of attack based on finite-time extended state observer. Nonlinear Dyn..

[27] Feng Z, Fei J (2018). Super-twisting sliding mode control for micro gyroscope based on RBF neural network. IEEE Access.

[28] Alharbi YM (2020). Super twisting fractional order energy management control for a smart university system integrated dc micro-grid. IEEE Access.

[29] Zhang M, Huang J, Cao Y, Xiong C-H, Mohammed S (2022). Echo state network-enhanced super-twisting control of passive gait training exoskeleton driven by pneumatic muscles. IEEE/ASME Trans. Mechatron..

[30] Li X, Liu J, Yin Y, Zhao K (2023). Improved super-twisting non-singular fast terminal sliding mode control of interior permanent magnet synchronous motor considering time-varying disturbance of the system. IEEE Access.

[31] Chen L (2023). Continuous adaptive fast terminal sliding mode-based speed regulation control of pmsm drive via improved super-twisting observer. IEEE Trans. Ind. Electron..

[32] Hou Q (2023). Super-twisting extended state observer based quasi-proportional-resonant controller for permanent magnet synchronous motor drive system. IEEE Trans. Transport. Electrific..

[33] Lee H, Utkin VI (2007). Chattering suppression methods in sliding mode control systems. Annu. Rev. Control..

[34] Wheeler G, Su C-Y, Stepanenko Y (1998). A sliding mode controller with improved adaptation laws for the upper bounds on the norm of uncertainties. Automatica.

[35] Huang Y, Kuo T, Chang S (2008). Adaptive sliding-mode control for nonlinear systems with uncertain parameters. IEEE Trans. Syst. Man Cybernet.-Part B: Cybernet..

[36] Plestan F, Shtessel Y, Bregeault V, Poznyak A (2010). New methodologies for adaptive sliding mode control. Int. J. Control.

[37] Utkin VI, Poznyak AS (2013). Adaptive siding mode control with application to super-twist algorithm: Equivalent control method. Automatica.

[38] Li Y, Liu A, Yang C (2019). Adaptive sliding mode control of a class of fractional-order nonlinear systems with input uncertainties. IEEE Access.

[39] Zhu J, Khayati K (2016). Adaptive sliding mode control—convergence and gain boundedness revisited. Int. J. Control.

[40] Fallaha CJ, Saad M, Kanaan HY, Al-Haddad K (2011). Sliding-mode robot control with exponential reaching law. IEEE Trans. Industr. Electron..

[41] Ma H, Wu J, Xiong Z (2017). A novel exponential reaching law of discrete-time sliding-mode control. IEEE Trans. Industr. Electron..

[42] Yang Z, Zhang D, Sun X, Ye X (2018). Adaptive exponential sliding mode control for a bearingless induction motor based on a disturbance observer. IEEE Access.

[43] Chen G, Deng F, Yang Y (2021). Practical finite-time stability of switched nonlinear time-varying systems based on initial state-dependent dwell time methods. Nonlinear Anal. Hybrid Syst.

[44] Khayati K (2015). Multivariable adaptive sliding-mode observer-based control for mechanical systems. Can. J. Electr. Comput. Eng..

[45] Lochan K, Singh JP, Roy BK, Subudhi B (2018). Adaptive time-varying super-twisting global SMC for projective synchronisation of flexible manipulator. Nonlinear Dyn..

[46] Mazhar, S., Khan, R., Malik, F. M., Saeed, A. & Ullah, H. Finite settling time control of nonlinear systems in presence of matched perturbations using barrier lyapunov function. In *2021 International Conference on Robotics and Automation in Industry (ICRAI)* 1–7. 10.1109/ICRAI54018.2021.9651447 (2021).

[47] Zhu J, Khayati K (2017). On new adaptive sliding mode control for MIMO nonlinear systems with uncertainties of unknown bounds. Int. J. Robust Nonlinear Control.

[48] Tilbury, D. *et al*. (2023, accessed 8 Dec 2023) Inverted Pendulum: Pid Controller Design.

